# Genetic association of intelligence with longevity in *Drosophila melanogaster*

**DOI:** 10.1371/journal.pone.0325154

**Published:** 2025-07-02

**Authors:** Mousumee Khan, Enkhchimeg Lkhagva, Hae-mi Kim, Chongkai Zhai, Sharif Hasan Siddiqui, Seong-Tshool Hong

**Affiliations:** 1 Department of Biomedical Sciences and Institute for Medical Science, Chonbuk National University Medical School, Jeonju, Chonbuk, South Korea; 2 Department of Ophthalmology, Visual and Anatomical Sciences, Wayne State University School of Medicine, Detroit, Michigan, United States of America; 3 Animal Diseases and Public Health Engineering Research Centre of Henan Province, Luoyang Polytechnic, Luoyang, China; 4 Department of Physiology, Wayne State University, Detroit, Michigan, United States of America; 5 John D. Dingell VA Medical Center, Detroit, Michigan, United States of America; BSRC Alexander Fleming: Biomedical Sciences Research Center Alexander Fleming, GREECE

## Abstract

Epidemiological studies in different populations, in different countries, and in different epochs consistently showed that high intelligence is positively correlated with longevity. The link between high intelligence and longevity has remained unknown, only to be assumed as a consequence of the socioeconomic difference associated with intelligence in human population. Here, we report that genome stability contributes both to lifespan and intelligence in *Drosophila melanogaster*. The intelligence of the genetically heterogenous flies was determined by T-maze olfactory memory assay, and the flies moving to the right direction defined as intelligent flies (INT) were separated from the flies moving to the wrong direction defined as non-intelligent flies (NINT). INT male and female lived 26.40% and 21.35% longer than NINT male and female, respectively, suggesting a possible genetic linkage between intelligence and longevity. The bidirectional selective breeding based on intelligence extended lifespans gradually generation by generation in INT breeding contrast to the reversed pattern in NINT breeding. INT of F12 generation lived longer than NINT of F12 generation, 63.91% for male and 67.88% for female, as a result from slower aging. The whole-genome transcriptome analysis showed the activation of the genes in ribosome and autophagy in INT and the pathways of genome stability and immune reaction in NINT. Especially, the genetic pathway associated with genome stability was most noticeable, indicating that genome stability contributes both to lifespan and intelligence in *D. melanogaster*.

## Introduction

A strong correlation between higher intelligence test (IQ) scores and longevity has been one of the most curious scientific questions, which led to the recent emergence of a new scientific field, cognitive epidemiology. Many different epidemiological studies have consistently found that IQ measured in early childhood predicted substantial differences in adult morbidity and mortality: higher IQ predicts longer lifespan [1–5]. Also, mortality rates about twice as high were observed in individuals with low IQ at age 11 as in those with high IQ (3.4% vs 1.7%) [6]. The strong association of intelligence-lifespan has been repeatedly observed in different populations, in different countries, and in different epochs [7–12]. A meta-analysis found a 24% increase in lifespan for people with 15 point-elevated IQ scores in both genders [13]. It should be noted that this study using the *Drosophila* model was focused on a specific aspect of intelligence—the relationship between longevity and cognitive ability, referring to a narrow aspect of intelligence rather than intelligence as a whole.

Although positive intelligence-lifespan association becomes an undeniable scientific fact, the reason of the association remains unknown until now. Currently, the most prevailing opinion is that either socioeconomic status influences both intelligence and health or higher intelligence leads to higher socioeconomic status, resulting in better life habits to achieve longevity [14–16]. The intelligence-lifespan association resulting from socioeconomic status seems to be plausible. However, observation on monozygotic twin weakened the socioeconomic hypothesis on the linkage between higher intelligence and longevity. Monozygotic twins adopted on the different families in different countries demonstrated identical IQs and lifespans within the twins [17,18], suggesting that genes determined the linkage of higher IQ and longevity.

Despite the possibility of a genetic relation between higher intelligence and longevity, there is not any report showing association of longevity with higher intelligence at genetic levels. It has been reported that longevity is associated with the lipoprotein pathway, translational processes, energy metabolism, and DNA repair [19]. However, an association of these pathways with intelligence has not been observed. Rather, the pathways of cholinergic metabolism, cell development, neural development, and synaptic regulation were reported to be associated with intelligence [20–22]. Although a common pathway governing intelligence and longevity has not been identified, a recent study using well defined genetically informative twins suggested that link between intelligence and longevity would be mostly genetic [17]. Understanding of a common genetic pathway governing longevity and intelligence would contribute to advance the perception of biological sciences as well as sociology. In this work, we found that the genetic pathway governing genome stability is the common pathway for longevity and intelligence in *D. melanogaster*.

## Materials and methods

### Fly strains and maintenance

The experimental design of the study is illustrated in S1 Fig. *D. melanogaster* strains used in this study are listed in S1 Table. The flies were maintained on standard cornmeal–agar–molasses hard medium under a 12-h light: 12-h dark cycle with 60% relative humidity at 25 °C with the addition of a few grams of *Drosophila* instant food plain (Hansol, DIFP-31020) on the surface of the standard food, except for laying eggs which was kept on standard cornmeal–agar–molasses soft medium by adding very small amounts of dried yeast on the top of the food. The composition of both soft and hard medium is described in S2 Table.

### T-maze olfactory memory assay

Two-day-old flies were used for the T-maze olfactory memory assay. Experiments were conducted in a dark room under the exposure of red light. Flies were collected to fresh food vials 30 min prior to conditioning for equilibration to the experimental room conditions of 25 °C temperature and 60% humidity [23]. For training, a total of 300 flies, 150 males and 150 females each, were transferred into a customized T-maze tube where they received the following sequence of stimulus: 1 min of an odor paired with 80 V electric shock (conditioning stimulus, CS^+^), 2 min of rest and 1 min of a second odor unpaired with electric shock (CS^-^). We used molasses (only) as a first odor paired with electric shock and DJ rice media without containing molasses as a second odor unpaired with electric shock. The trained flies were kept to a food vial to be tested at 30-min time point (considered as a short-term smelling test) or 24-hour time point (considered as a long-term smelling test) after the training period. For the long-term odor memory test, flies needed to complete 5 cycles of training in a T-maze tube with a 15-min interval. During the short-term and long-term smelling test, flies were allowed to move for 1 min to choose between a T-maze tube exposed with the CS^+^ odor and another T-maze tube exposed with the CS^-^ odor. Flies that chose the T-maze tube exposed with CS^+^ odor were considered as a non-intelligent (NINT) group. On the other hand, flies that chose the T-maze tube exposed with CS^-^ odor were considered as intelligent (INT) group. Only the intelligent flies who passed the short-term smelling test were selected for the long-term smelling test. The flies that passed the long-term smelling test were again trained into a customized T-maze tube in which a CS^+^ odor was exposed with red light and a CS^-^ odor was exposed with yellow light. Except for the addition of two different lights on two different sides of a T-maze tube, the training conditions were the same as before. After 30 min of training, the trained flies were used for a short-term coloring test with odors. Flies that chose a CS^-^ odor exposed with yellow light were selected to train for a long-term (24 hours later) coloring test with odors. Finally, the flies that passed the long-term coloring test with odors were considered as intelligent and were selected for crossing to generate a new generation. For all memory assays, flies were divided into two sub-groups where one sub-group was trained with molasses as the conditioning stimulus paired with electric shock (CS^+^) and DJ rice media unpaired with electric shock (CS^-^) while the other sub-group was trained with DJ rice media as CS^+^ and molasses as CS^-^. This method removes any bias of flies having a high preference for one odor. Each group was tested and performance index (PI) for each group was calculated by the following formula-


$$\begin{array}{c} \frac{\text{1}}{\text{2}}\text{ PI}=\frac{\#{\text{\textbackslash{} CS}}^{-}\text{flies}-\#{\text{\textbackslash{} CS}}^{+}\text{flies}}{\#\text{total}flies} \end{array}$$ (1)

The final PI was measured by averaging the two half PIs of each experimental group [24,25].

### Lifespan assay

Lifespan assays were performed as described previously [26–28]. Briefly, 150 adult male and 150 adult female flies were used for the lifespan assay. Flies were flipped into fresh food every 2 days and the number of deaths was scored.

### Startle-induced negative geotaxis assay

Startle-induced negative geotaxis assay was performed on the 5^th^, 30^th^, 60^th^ and 90^th^ days post-eclosion as previously described [26,29]. In brief, 15 flies per genotype were anesthetized with CO_2_, kept in a 15 mL of conical tube and bunged with cotton wool. The flies were allowed to recover for 30 min prior to the assay. The tube was gently tapped for a few seconds to gather the flies at the bottom of the tube. The experimental flies were allowed to climb for 1 min to observe their climbing ability. After 1 min, the number of flies at the top (above the 10 mL line) and at the bottom (below the 2 mL line) were recorded. Three trials were performed at each time point. The performance index (PI) was calculated for 15 flies of each genotype (average of 3 trials) using the formula PI = 0.5 × (n_total_ + n_top_ − n_bottom_)/n_total_, where n_total_ is the total number of flies, n_top_ is the total number of flies at the top, and n_bottom_ is the total number of flies at the bottom.

### Measuring body weights

The body weights were measured on the 5^th^, 30^th^, 60^th^, and 90^th^ days post-eclosion as described previously [26,27]. 15 adult flies from each genotype were sedated with CO_2_ plate and weighed immediately. For each group, three independent replicates were averaged.

### Eye imaging by light microscopy

Adult flies were collected on the 5^th^, 30^th^, 60^th^ and 90^th^ days post-eclosion as previously described [26,29,30]. Briefly, 15 flies per genotype were immobilized by freezing at −80 °C for 3 ~ 4 hr before taking light microscopic images of eyes. Eye images were observed under an AmScope 6.7X to 45X Boom Stereo Dissecting Microscope, equipped with AmScope Microscope Eyepiece Camera (AmScope, MU1000) and analyzed using the ImageJ software. Disrupted ommatidia area was considered as an area containing two or three fused ommatidia.

### Histological examination

Histological examination was carried out on the 5^th^, 30^th^, 60^th^ and 90^th^ days post-eclosion. 15 adult flies per genotype were prepared by freezing at −80 °C for 3 ~ 4 hr and fixed in 10% neutral-buffered formalin, followed by embedding in paraffin and sectioning at 4 ~ 6 μm to be placed on glass microscopic slides. After removing paraffin from the sections on the microscopic slides with hot water, the slides were air-dried and baked at 65 °C (overnight). All tissue sections were stained with Hematoxylin and Eosin (H&E) following standard staining procedure [26,29,31,32]. The stained tissue images were obtained on a light microscope (AmScope, T690C-PL) at magnification of 100X, equipped with a digital microscopic camera (AmScope, MU-1803). Vacuoles and damaged tissue area were analyzed using ImageJ software.

### Scoring of the neurodegeneration index

Neurodegeneration is indicated by the presence of vacuoles in the brain neuropil. The H&E-stained brain tissues were analyzed and vacuoles were quantified. Five levels of neurodegeneration (0, 1, 2, 3, 4 and 5) were considered: 0, 1 normal to low; 2, 3 moderates; 4, 5 strong to severe (S3 Fig). The scoring of neurodegeneration was done blindly with respect to genotype and/or three groups in the study.

### RNA isolation and quality control

Total RNA was isolated from 5-days-old INT and NINT *D. melanogaster* at the F_12_ generation and F_0_ flies using Trizol reagent (Invitrogen) according to the manufacturer’s protocol. RNA quality was assessed by Agilent 2100 bioanalyzer using the RNA 6000 Nano Chip (Agilent Technologies, Amstelveen, The Netherlands) (S8a-d Fig, S3 and S4 Tables) and RNA quantification was performed using ND-2000 Spectrophotometer (Thermo Inc., DE, USA).

### Library preparation and mRNA sequencing

Libraries were prepared from total RNA using the SMARTer Stranded RNA-Seq Kit (Clontech Laboratories, Inc., USA). The isolation of mRNA was performed using Poly (A) RNA Selection Kit (LEXOGEN, Inc., Austria). The isolated mRNAs were used for the cDNA synthesis and shearing following manufacture’s instruction. Indexing was performed using the Illumina indexes 1–12. The enrichment step was carried out using PCR. Subsequently, libraries were checked using the Agilent 2100 Bioanalyzer (DNA High Sensitivity Kit) to evaluate the mean fragment size. Quantification was performed using the library quantification kit using a StepOne RealTime PCR System (Life Technologies, Inc., USA). High-throughput sequencing was performed as paired-end 100 sequencing using HiSeq 2500 (Illumina, Inc., USA). mRNA-Seq reads were mapped using TopHat software [33] tool in order to obtain the alignment file. DEGs (Differentially expressed genes) were determined based on counts from unique and multiple alignments using coverage in Bedtools [34]. The overall experimental design for the mRNA sequencing analysis is illustrated in S9 Fig.

### mRNA-seq data analysis

The read count data were processed based on geometric median and ratio normalization method using DESeq2 package [35] within R program [36]. All the read count was imported into DESeq2 and read counts were log transformed to validate the data quality by the hierarchical clustering method. All normalized counts were used for further analysis. DEG’s were identified by Wald test in DESeq2 package by using three pair of group comparison: 1) INT-F_0_, 2) NINT-F_0_, 3) INT-NINT, filter criterion was an adjusted *P* value <0.01. To better understand the functional involvements of the DEGs, the latest Ensembl release annotation set of *D. melanogaster* (AH89395) was used for gene level annotation in AnnotationHub package [37]. Gene ontology (GO) terms and Kyoto Encyclopedia of Genes and Genomes (KEGG) associated with the significant genes were performed by the clusterProfiler package [38] with cut-off values as adjusted *P* value < 0.05. The enrichplot package [39] was used for visualization of Gene Set Enrichment Analysis (GSEA). Volcano plot associated with the significant genes were visualized by the ggvenn package [40] with cut-off values as adjusted *P* value < 0.05, −2 < Log2fold change >2. Venn diagram was visualized by ggplot2 package [41] for GO and KEGG pathways.

### Statistical analysis

Lifespan data was analyzed using the Kaplan–Meier method and data was prepared using GraphPad Software Prism version 7.04. The genetic association between intelligence and lifespan (both maximum and average lifespan) were analyzed by the linear regression method. Statistical analyses were performed using unpaired Student’s t-test and one-way ANOVA with multiple comparisons test using SAS version 9.4 (SAS Institute Inc., Cary, NC, USA). *P* values are indicated as asterisks highlighting the significance of comparisons: *P < 0.05; **P < 0.01; ***P < 0.001.

## Results and discussion

### Higher intelligence was associated with longer lifespan in *D. melanogaster*

It has been widely observed in animal kingdom that the lifespan of animal species is positively correlated with intelligence within phylogenetically related animal species [42–44]. Considering the correlation, we speculated a determinant role of genes on cognitive epidemiological observations. We tested the hypothesis by analyzing the relationship between the lifespan and intelligence of *D. melanogaster*. A genetically heterogenous population of *D. melanogaster* was established by selectively breeding 7 different strains of the flies for 4 generations. The intelligence of the heterogenous flies was determined by T-maze olfactory memory assay that evaluated the memory of two different odors at the two opposite sides in the T-maze tube after 30 min of training. *D. melanogaster* moving to the right direction were defined as intelligent flies (INT) while flies moving to the wrong direction were defined as non-intelligent flies (NINT).

**Fig 1:**
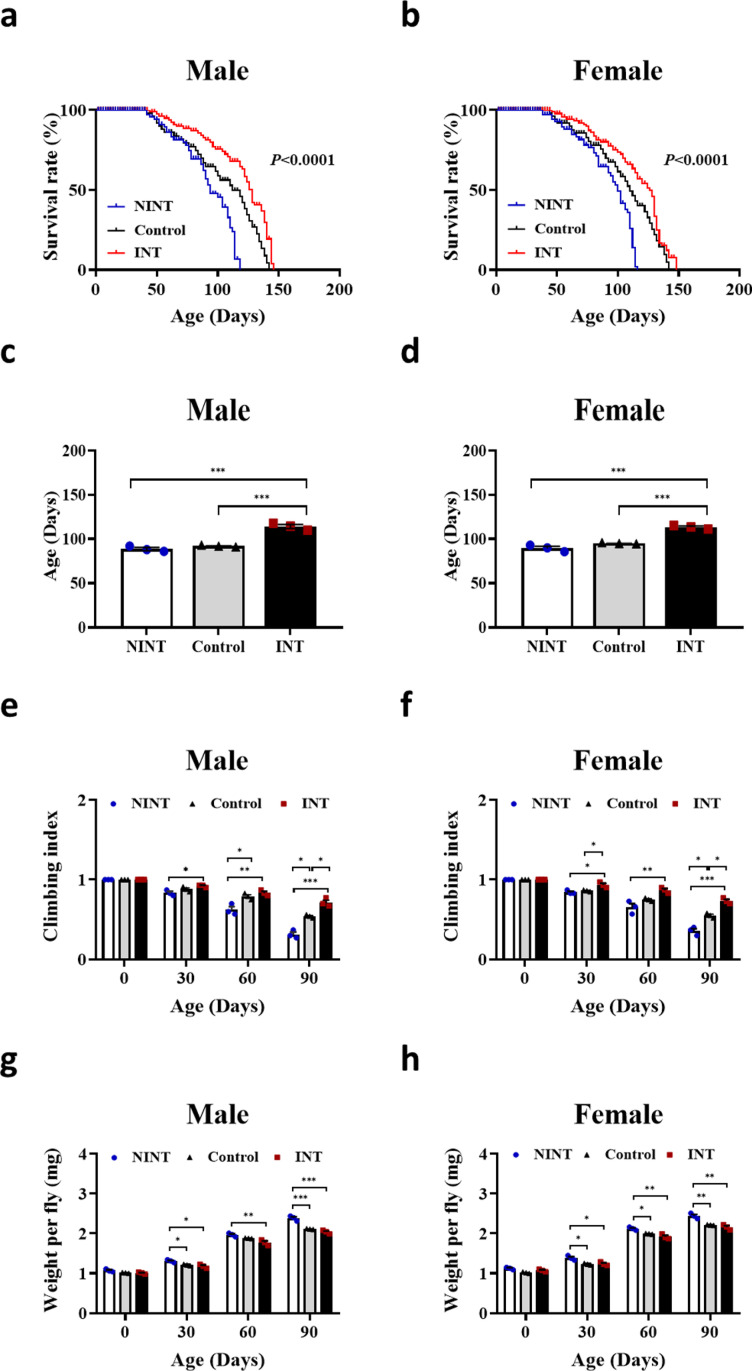
The lifespans of *D. melanogaster* were positively correlated with the level of their intelligence. **a-b** Lifespan analysis of male and female *D. melanogaster*, using Kaplan-Meier survival method. **c-d** The average lifespan of male and female *D. melanogaster*. **e-f** The startle-induced negative geotaxis assay of male and female *D. melanogaster* during age progression. **g-h** The body weights of male and female *D. melanogaster* with age progression. All data are are shown as mean ± SE. *P < 0.05, **P < 0.01 and ***P < 0.001.

After separating the flies into two groups by 4 generations of selection, the lifespans of both INT and NINT were monitored. The maximum lifespan of the INT was also longer than that of NINT by 28% in male and 32% in female flies (Fig 1a-b). The average lifespans of the INT flies were longer than those of NINT flies, 26.40% in male and 21.35% in female groups (Fig 1c-d). The lifespan difference between INT and NINT was comparable to that of the human population in which people with higher IQs tend to live longer compared to those with lower IQs [2,26,27] indicating a general genetic linkage of higher intelligence with longevity in the animal kingdom. In addition to lifespan, INT maintained physical strength during aging process than NINT as evident by a climbing assay (Fig 1e-f). Also, the body weight of INT increased much slower than that of NINT (Fig 1g-h). The physical strength and body weight differences further validated the slower aging of INT.

### Higher intelligent flies aged slower than nonintelligent flies

**Fig 2:**
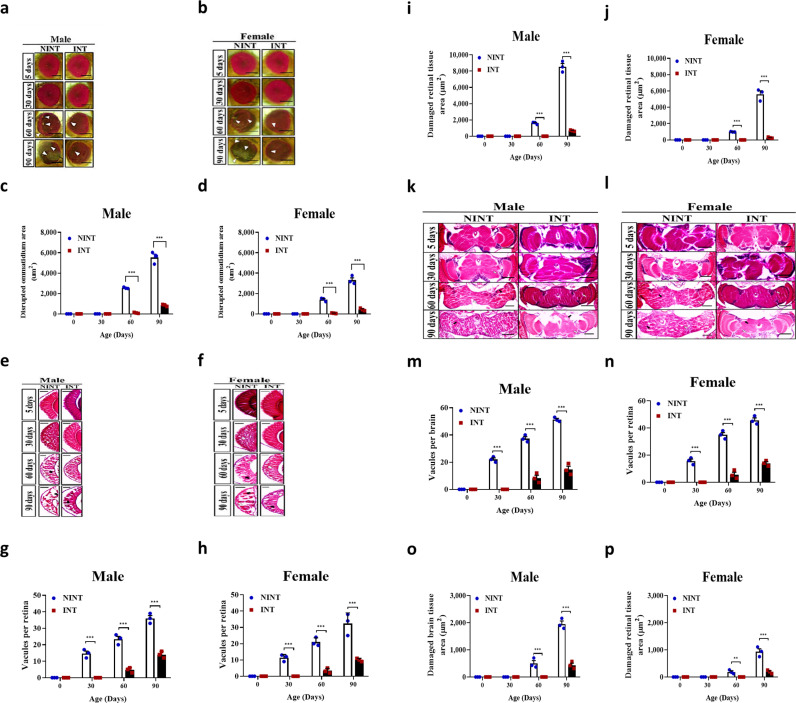
The intelligent flies aged slower than the nonintelligent flies. **a-b** The representative light microscopic images of the eyes of male and female *D. melanogaster* during age progression. Eye damages are indicated as white arrows. Scale bar, 50 µm. **c-d** Quantification of the disrupted ommatidium area of male and female *D. melanogaster* during age progression. **e-f** The representative images of the H&E-stained histological sections of the retina of male and female *D. melanogaster* during age progression. Vacuoles and damaged retinal tissue area are indicated as white arrows and black arrows, respectively. Scale bar, 30 µm. **g-h** Quantification of vacuoles per retina of male and female *D. melanogaster* during age progression. **i-j** Quantification of the damaged retinal tissue area of male and female *D. melanogaster* during age progression. **k-l** The representative images of the H&E-stained histological sections of the brain of male and female *D. melanogaster* during age progression. Vacuoles and damaged brain tissue area are indicated as white arrows and black arrows, respectively. Scale bar, 50 µm. **m-n** Quantification of vacuoles per brain of male and *D. melanogaster* during age progression. **o-p** Quantification of the damaged brain tissue area of male and female *D. melanogaster* during age progression. All data are shown as mean ± SE. *P < 0.05, **P < 0.01 and ***P < 0.001.

Assessment of aging by visual observation [26,31,45] further confirmed that the INT flies aged more slowly compared to the NINT flies. There was no obvious morphological defect found in both male and female flies of INT and NINT until the 30^th^ day of age (Fig 2a-b). However, loss of pigmentation as well as ommatidia disruption became observable starting from the 60^th^ day in the NINT flies. The eye damage of NINT became more prominent as flies aged while the age-related eye damage was significantly less observable in INT. The disrupted areas of ommatidia were 847 ± 59 µm^2^ for male and 429.33 ± 72.38 µm^2^ for female at 90^th^ day in INT, which were very significantly smaller than 5531.33 ± 338.95 µm^2^ for male and 3311.33 ± 233.49 µm^2^ for female in NINT (Fig 2c-d).

Histological examination on the eyes confirmed faster aging in NINT and slower aging in INT (Fig 2e-f). The fast-aging became obvious event at the 30-days-old flies of NINT as evident by vacuolar changes (Fig 2g-h). The retinal degeneration of NINT was exacerbated as the flies got older while there was much slower degeneration of the eyes of INT (Fig 2i-j). The whole brain morphology also indicated a much slower aging in INT (Fig 2k-l). The H&E brain section showed an age-dependent heavy neurodegeneration in NINT (Fig 2m-p). Overall, these histological examinations confirmed that higher intelligent flies aged slower than nonintelligent flies.

### A bidirectional selective breeding on intelligence over 12 generations confirmed a genetic association between higher intelligence and longevity in *D. melanogaster*

**Fig 3:**
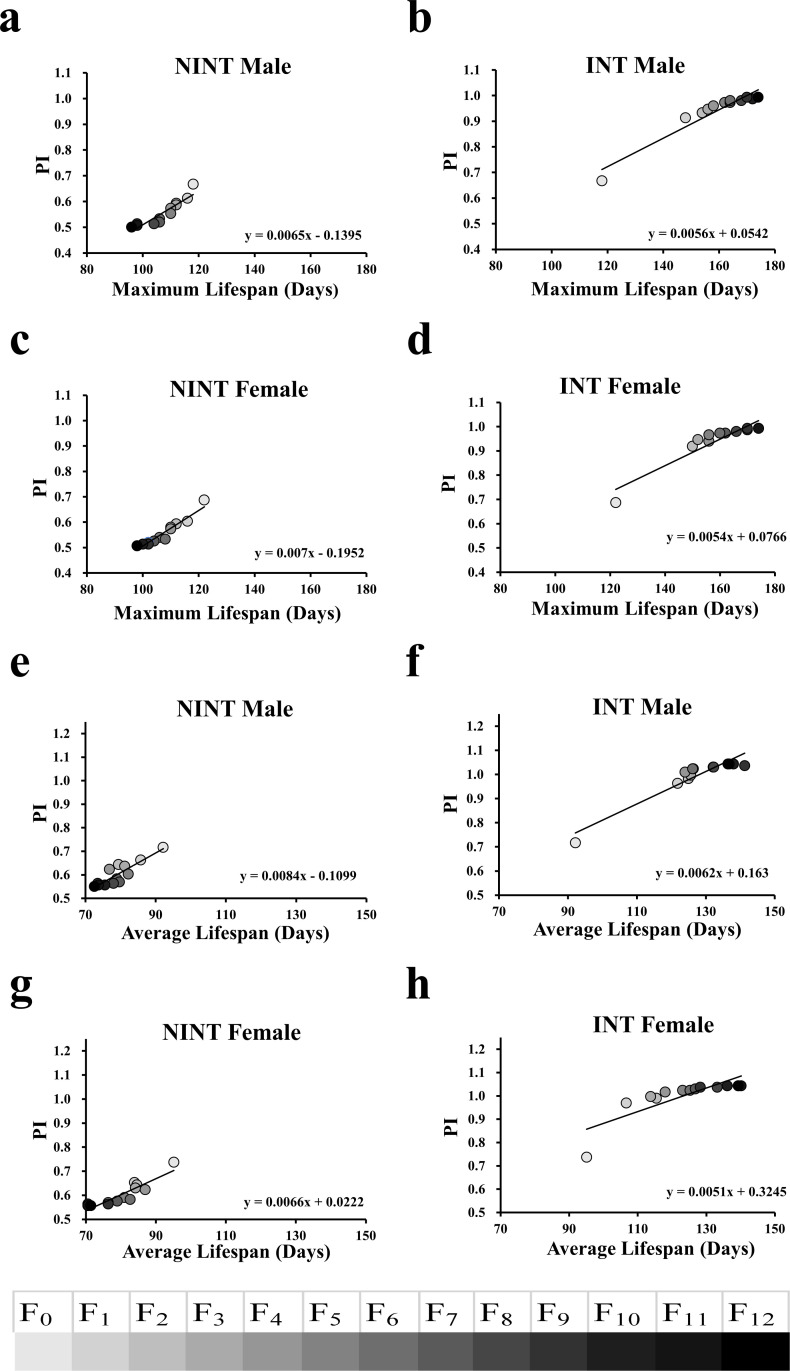
The bidirectional selective breeding of intelligence indicated a genetic association between intelligence and lifespan in *D. melanogaster.* **a-b** Linear regression between PI and the maximum lifespan of the NINT and INT male *D. melanogaster*. **c-d** Linear regression between PI and the maximum lifespan of the NINT and INT female *D. melanogaster*. **e-f** Linear regression between PI and the average lifespan of the NINT and INT male *D. melanogaster*. **g-h** Linear regression between PI and the average lifespan of the INT and INT female *D. melanogaster*.

If longevity and higher intelligence are genetically linked, selection to intelligence should extend the lifespan too. We tested this hypothesis by a bidirectional selective breeding for intelligence for 12 generations, in which INT bred with INT and NINT bred with NINT for 12 generations. The INT and NINT flies were distinguished by the fly olfactory memory test as in the case of Fig 1. The percentage of flies choosing the correct direction in the T-maze test was represented as performance index (PI). Selective breeding for intelligence consecutively increased PI for 12 generations while selective breeding against intelligence decreased PI gradually (Fig 3). The PI values were increased from 0.667 (male) and 0.687 (female) at F_0_ generation to 0.993 (male) and 0.993 (female) at F_12_ generation, respectively. Meanwhile, the selective breeding against intelligence, however, decreased PI values to 0.500 (male) and 0.507 (female) at F_12_ generation.

The lifespans of the flies were increased continuously generation by generation along with the intelligence increase by the intelligence selection. On the contrary, selection against intelligence decreased the lifespan continuously along with the intelligence decrease (Fig 3 and S2a-d Fig). The average lifespans of the F_12_ generation were increased by 44.29% (male) and 44.16% (female) compared to F_0_ by selective breeding for intelligence but decreased by 19.613% (male) and 23.72% (female) by selective breeding against intelligence. Similarly, the maximum lifespans of F_12_ were increased by 56% (male) and 52% (female) compared to F_0_ by selective breeding for intelligence but decreased by 22% (male) and 24% (female) by selective breeding against intelligence. Overall, the average lifespan differences between INT and NINT at F_12_ were 63.91% in male and 67.88% in female. These results clearly indicate that intelligence and longevity are connected in *D. melanogaster*.

The genetic association between intelligence and longevity was further validated by statistic methods. Linear regression analysis showed a definite linear relationship between intelligence and maximum lifespan (Fig 3a-d) as well as intelligence and average lifespan (Fig 3e-h). The coefficients of determination were in the range of 0.6658 ~ 0.9252 with the *P* values of <0.0001 ~ 0.0007 in each of the association analyses (Fig 3), proving that longevity and higher intelligence are genetically associated in *D. melanogaster*.

### The genetically intelligent flies aged slowly while the genetically non-intelligent flies aged quickly

**Fig 4:**
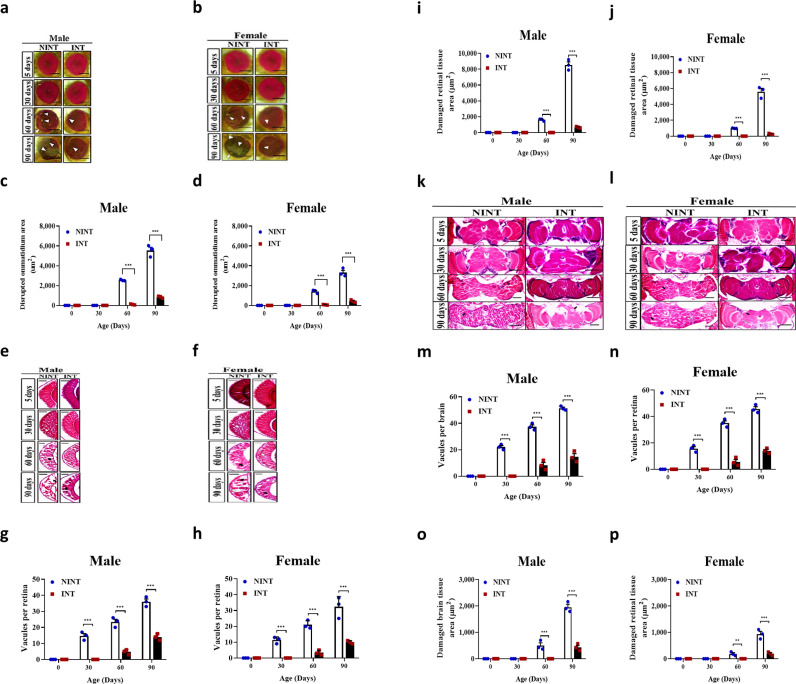
The genetically intelligent flies aged slowly while the genetically non-intelligent flies aged quickly. **a-b** The representative light microscopic images of the eyes of male and female *D. melanogaster* during age progression. Eye damages are indicated as white arrows. Scale bar, 50 µm. **c-d** Quantification of the disrupted ommatidium area of male and female *D. melanogaster* during age progression. **e-f** The representative images of the H&E-stained histological sections of the retina of male and female *D. melanogaster* during age progression. Vacuoles and damaged retinal tissue area are indicated as white arrows and black arrows, respectively. Scale bar, 30 µm. **g-h** Quantification of vacuoles per retina of male and female *D. melanogaster* during age progression. **i-j** Quantification of the damaged retinal tissue area of male and female *D. melanogaster* during age progression. **k-l** The representative images of the H&E-stained histological sections of the brain of male and female *D. melanogaster* during age progression. Vacuoles and damaged brain tissue area are indicated as white arrows and black arrows, respectively. Scale bar, 50 µm. **m-n** Quantification of vacuoles per brain of male and female *D. melanogaster* during age progression. **o-p** Quantification of the damaged brain tissue area of male and female *D. melanogaster* during age progression. All data are shown as mean ± SE. *P < 0.05, **P < 0.01 and ***P < 0.001.

It is possible that the extended lifespan of intelligent flies could be a result from slow aging or other factors. We investigated this possibility by observing eye degeneration, retinal degeneration, locomotor impairment, flight muscle tissue degeneration, neurodegeneration, and body weight changes during the age progression of the fly populations. Ommatidia started to be disrupted from the 30^th^ day in the NINT flies while the disruption was not detected until the 60^th^ day in the INT and F_0_ flies (Fig 4a-b). At the 90^th^ day, the disrupted areas of ommatidia were the smallest among INT flies (373 ± 36.86 µm^2^ in male and 229.67 ± 52.76 µm^2^ in female), which were significantly smaller than those of the F_0_ flies (1311.67 ± 73.05 µm^2^ for male and 1077.67 ± 82.83 µm^2^ for female) and the NINT flies (6201.67 ± 500.78 µm^2^ for male and 2358.67 ± 186.47 µm^2^ for female) (Fig 4c-d).

In accordance with the age-dependent disruption of ommatidia, retina, brain, and flight muscle were also degenerated similarly (Fig 4 and S4 and S5 Fig). The age-dependent vacuoles in the retina and the brain were observed starting from the 30^th^ day of age in both NINT and F_0_ flies in contrast to the 60^th^ day of age in the INT flies (Fig 4e-p and S4 Fig). The vacuolated areas in the retina and brain due to the age-dependent tissue degeneration were very different among F_0_, INT, and NINT flies on the 90^th^ day (Fig 4i, o and S4 Fig). The degenerated areas in the retina were least in INT flies, (654.67 ± 52.57 µm^2^ for male and 251.33 ± 49.27 µm^2^ for female), being significantly smaller than those in the F_0_ flies (5753.67 ± 195.73 µm^2^ for male and 2741.67 ± 162.65 µm^2^ for female) and dramatically smaller than the NINT flies (9221.67 ± 551.750 µm^2^ for male and 5335.67 ± 524.21 µm^2^ for female) (Fig 4i). Also, the degenerated areas in the brains were smallest in INT flies (447 ± 50.56 µm^2^ for male and 177.33 ± 20.76 µm^2^ for female), being significantly smaller than the F_0_ flies (842.33 ± 80.45 µm^2^ for male and 471.67 ± 56.13 µm^2^ for female) and dramatically smaller than the NINT flies (1987.33 ± 65.41 µm^2^ for male and 903 ± 97.44 µm^2^ for female) (Fig 4o). The integrities of flight muscle tissue were also maintained in a youthful state at the 90^th^ day of age in the INT flies unlike the F_0_ and NINT flies (S5a-d Fig).

The locomotive test by using startle-induced negative geotaxis assay further confirmed that aging progress was even further slowed in INT than NINT and F_0_ flies. The male and female flies of INT climbed the test tube 1.67 and 1.41 times faster than the F_0_ flies and 2.62 and 2.31 times faster than the NINT flies on the 90^th^ day (S6 Fig). The body weights of INT were 2.01 ± 0.03 mg in male and 2.08 ± 0.02 mg in female, being leaner than the F_0_ flies (2.25 ± 0.03 mg in male and 2.34 ± 0.03 mg in female) and the NINT flies (2.27 ± 0.04 mg in male and 2.38 ± 0.03 mg in female) on the 90^th^ day (S7 Fig). Considering that increment of body weight during age progression is a universal phenomenon in animal kingdom [46–48], the group difference of body weight also validated a slower aging of INT.

### A whole genome expression analysis proved that the intelligent flies were genetically different from the non-intelligent flies

**Fig 5:**
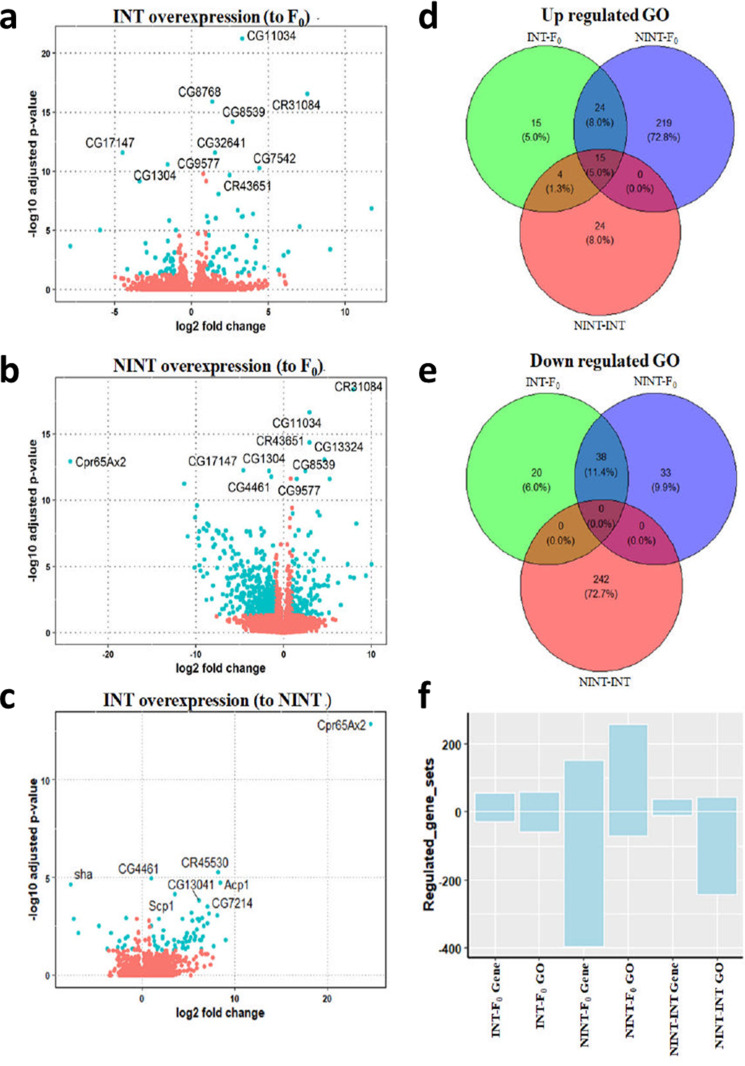
INT, NINT, and F_0_ flies were genetically different each other. **a-c** The volcano plot for the DEGs. The most significantly different top 10 genes are presented in the volcano plots. **d-e** Venn diagram showing the up- and down-regulated GO terms in each group; INT-F_0_: INT compared to F_0_, NINT-F_0_: NINT compared to F_0_, and NINT- INT: INT compared to NINT. **f** Up- and down- regulated DEGs and GO terms in each comparison group; INT-F_0_: INT compared to F_0_, NINT-F_0_: NINT compared to F_0_, and NINT- INT: INT compared to NINT.

In order to further investigate the genetic evidence for the association between higher intelligence and longevity, a transcriptome analysis was performed by sequencing the total mRNA. A principal component analysis (PCA) categorized for three comparison groups, INT-F_0_, NINT-F_0_, and INT-NINT showed separate clusters (S10 Fig), indicating that these three flies were genetically different each other. The differentially expressed gene analysis by a negative binomial test showed that changes in gene expression were relatively modest, and most genes had similar expression levels (adjusted *P* value <0.01) among the three groups of *D. melanogaster*. However, a subset of genes displayed remarkably altered expression where 56, 152, and 26 genes were significantly upregulated (adjusted *P* value <0.01, negative binomial test; S5 Table), and 30, 397, and 10 genes were downregulated (adjusted *P* value < 0.01, negative binomial test; S6 Table) in INT compared to F_0_, NINT compared to F_0_, and INT compared to NINT each. It should be noted that the genes of NINT were much more significantly disturbed compared to INT so that NINT has much more up- or down-regulated genes than INT. These genes, visualized in the volcano plot in Fig 5a-c, indicate significant genetic differences among the three groups of flies. These differences may suggest potential molecular pathways linking intelligence and longevity in Drosophila, though further investigation is required to establish causal relationships. Since INT and NINT were selected in the lab condition, the lab environment could affect both INT and NINT in the same direction. The twenty-four gene ontology (GO) terms were up-regulated, and 38 GO terms were down-regulated in both INT and NINT (Fig 5b, S8 and S9 Table), meaning that these GOs are associated with the lab environment.

The differentially expressed genes were grouped based on GO. Grouping of the differentially expressed genes by GO showed that the transcriptome was much more significantly perturbed in NINT than INT by the intelligence selection (Fig 5f). Small numbers of individual specific genes were differentially expressed in INT compared to F_0_ generation so that the numbers of genes and GO were almost same. While the number counted from the differentially expressed genes were reduced in counting differentially expressed GO pathways in NINT, which means that majority of the differentially expressed genes in NINT were associated in the pathways.

### The genetic factors responsible for longevity and intelligence were identified

**Fig 6:**
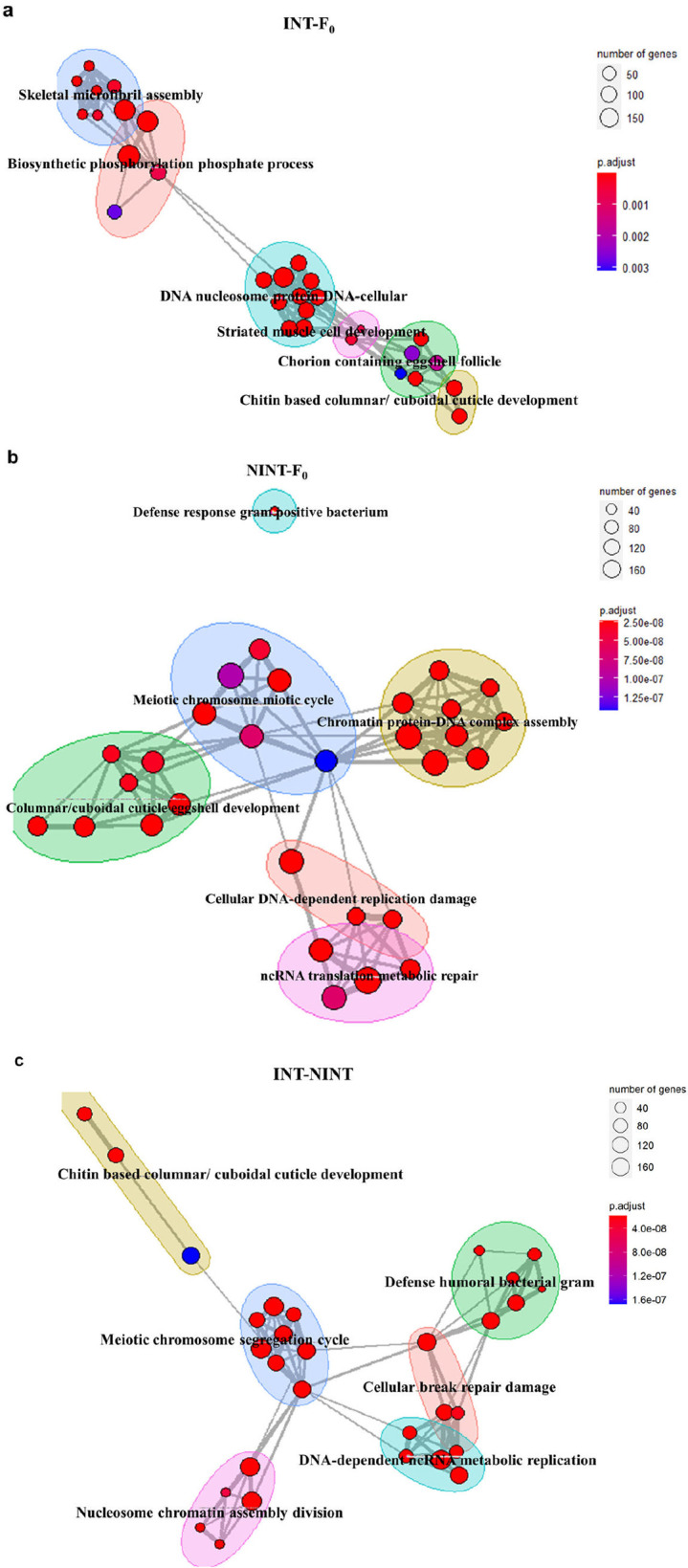
The genes involved in cellular homeostasis and genome stability played a role in the genetic linkage of intelligence and longevity. **a-c** Enrichment map of networks based on functionally enriched GO terms for each comparison group; INT-F_0_: INT compared to F_0_ **(a)**, NINT- F_0_: NINT compared to F_0_ **(b)** and INT-NINT: INT compared to NINT **(c)**.

The differentially expressed genes after the intelligent selection were analyzed by functional network analysis method. The genes of both INT and NINT compared to F_0_ were grouped into five different groups although connection patterns were different in INT and NINT (Fig 6). The 5 functional networks were the networks associated with cellular homeostasis or genome stability. The comparison of the gene network of INT with that of NINT further validated that the genes of cellular homeostasis (cuticle development, cellular break repair damage, humoral defense) and genome stability (meiotic chromosome segregation cycle, nucleosome chromatin assembly division, DNA-dependent ncRNA metabolic replication) were altered between INT and NINT.

**Fig 7:**
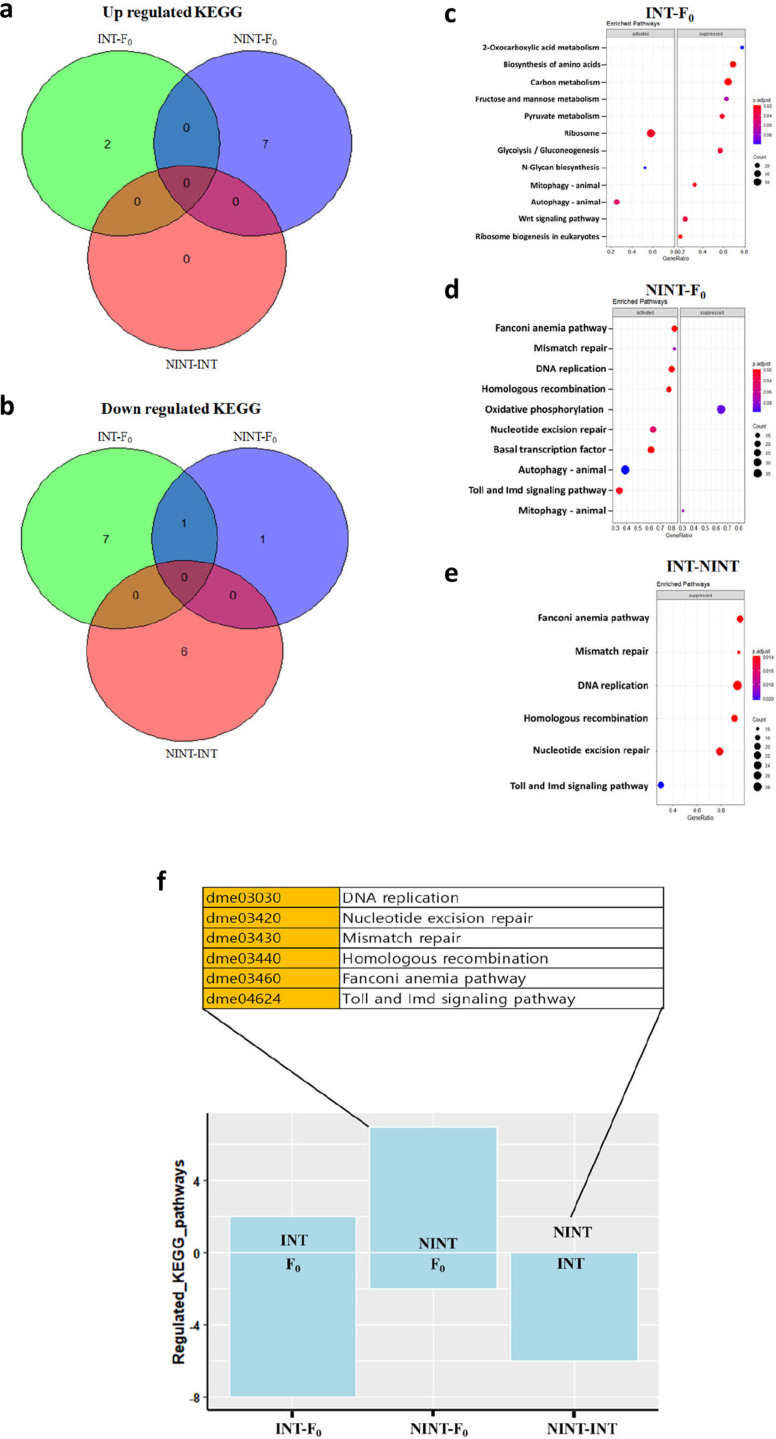
The genetic factors responsible for longevity and intelligence were identified. **a-b** Venn diagram showing the up- and down-regulated pathways of KEGG in each comparison group; INT-F_0_: INT compared to F_0_, NINT-F_0_: NINT compared to F_0_, and INT- NINT: INT compared to NINT. **c-e** Comparison of the differentially expressed pathways identified by KEGG pathway analysis for each comparison group; INT-F_0_: INT compared to F_0_, NINT-F_0_: NINT compared to F_0_, and INT- NINT: INT compared to NINT. **f** List of differentially expressed pathways identified by KEGG pathway analysis for each comparison group; INT-F_0_: INT compared to F_0_, NINT-F_0_: NINT compared to F_0_, and INT- NINT: INT compared to NINT.

The functions of the differentially expressed genes were analyzed at high levels by using Kyoto Encyclopedia of Genes and Genomes (KEGG) pathway enrichment program [49]. Since the KEGG pathway provides more defined pathways at high levels, the KEGG pathway visualized the more defined pathways than the GO analysis (Fig 7a-b). Compared to F_0_, the pathways of ribosome and autophagy were up-regulated in INT while the pathways related to metabolisms were down-regulated (Fig 7c-e). Meanwhile, the pathways associated with genome stability and immune reaction was upregulated and the pathway of oxidative phosphorylation responsible for ATP generation was down regulated in NINT. The KEGG comparison of INT with NINT, which eliminates genes associated with the environmental factors during the selection process, visualized more precise pathways associated with higher intelligence and longevity. The pathways related to genome stability (Fanconi anemia pathway, Mismatch repair, DNA replication, Homologous recombination, and Nucleotide excision repair) and immune reaction (Toll and lmd signaling pathway) were up-regulated in NINT, and these pathways were down-regulated in INT (Fig 7c-f). Although the upregulation of specific pathways in INT were not visible, the genes of CG4897, CG3712, CG8338, CG18492, and CG12559 which belong to the pathways of ribosome or autophagy were most noticeable (S7 Table).

## Conclusions

The comparative transcriptome analysis of the INT, NINT, and F_0_ flies indicated that the pathways of genome stability, immune reaction, metabolism, ribosome or autophagy, and cellular homeostasis control the common pathway of longevity and higher intelligence. An interference factor which is rooted in a lab environment during the selection procedure can affect both INT and NINT flies in the same direction. In fact, 24 GOs were up-regulated and 38 GOs were down-regulated in both INT and NINT (Fig 5d-e and S8 and S9 Table). Because INT and NINT selections were in opposite directions each other, these genes could be the genes associated with an interference factor. We eliminated the interference factor by comparing the genes between INT and NINT (Figs 5–7). Stringent high-level analysis by using the KEGG program^34^ and elimination of this interference factors visualized the activation of the genes in ribosome and autophagy in INT and the pathways of genome stability and immune reaction in NINT. Among these pathways, the genetic pathway associated with genome stability was most noticeable (Fig 6 and 7), indicating that genome stability contributes both to lifespan and intelligence in *D. melanogaster*. It seems that the pathways associated with genome stability were upregulated in NINT because their genomes were not stably maintained.

Collectively, these results well accord with the previous reports of positive association of the pathways of ribosome and autophagy with longevity [49] and negative association of genome instability [50,51] and activated immune reaction [52,53] with lifespan. Among the alteration of the expression levels of the genes, the pathways of genome stability were most notable (Fig 7c-f). Also, considering that the genes of ribosome and autophagy activated in INT are involved in maintaining cellular integrity [54,55], the genome stability and maintenance of cellular integrity seems to be the source of the genetic linkage of longevity and higher intelligence.

Stable maintenance of genome integrity and maintenance of cellular integrity are the most important and critical tasks for both germline and somatic cells [56]. Living organisms maintain their genome stability by an error-free replication of genetic material, the repair of replication mistakes or damaged genetic material, precise separation of genetic material during the formation of chromosomes, and precise chromosomal arrangement during meiosis and mitosis [57–59]. Genomic instability is associated with premature ageing [60], predisposition to various types of cancer [61], Ataxia telangiectasia [62], Nijmegen breakage syndrome [63], Fanconi anemia [64], and Cockayne’s syndrome [65]. Other than cancers, all of the diseases associated with genome instability are manifested by aging or neurological symptoms. Also, genome instability has long been implicated as the main determining factor for aging and health of individual organism in all eukaryotes [51,66–68]. The genome instability and the rate at which this instability occurs have been observed to increase with age [69,70]. The association of genome instability with aging or neurological symptoms indicates a possible association of genome stability with higher intelligence and longevity.

Moreover, we acknowledge that the relationship between cognitive performance and lifespan is multifactorial and likely governed by a complex interplay of genetic, epigenetic, and environmental factors. Although our findings suggest that certain genetic pathways—particularly those involved in genome stability, autophagy, and cellular maintenance—may contribute to both enhanced cognitive ability and extended longevity, causality remains unresolved. It is equally plausible that age-associated cognitive decline may stem from progressive neurodegenerative changes, including synaptic loss, diminished neurogenesis, impaired proteostasis, or declining sensory perception, rather than intrinsic genetic differences alone [71,72]. To distinguish these scenarios, future studies incorporating longitudinal behavioral assays, real-time imaging of neural circuitry, and transcriptomic profiling at single-fly resolution across the lifespan will be crucial [73,74]. In particular, longitudinal and single-cell resolution analyses may prove critical in dissecting the causal relationship between cognition and aging—distinguishing whether enhanced neural function actively mitigates systemic aging, or whether delayed emergence of aging hallmarks enables the preservation of cognitive capacity [75,76]. Such integrative approaches will be instrumental in elucidating whether the differentially expressed genes identified in this study function upstream as drivers, downstream as consequences, or in a feedback loop that coordinates the aging trajectory with cognitive resilience.

Although the positive correlation between intelligence and lifespan is well noted within human population [77,78], the same relationship has also been observed among various species in the animal kingdom. More intelligent animals tend to have longer lifespan within the phylogenetically related species as demonstrated in the case of whale [79], parrot [80], primate [42] etc. The genetic connection of higher intelligence with longevity within species and inter-species strongly suggest a genetic association between longevity and intelligence, denying the current prevailing opinion of the influential role of socioeconomic status within human population in lifespan. Nonetheless, we do not exclude the possibility that environmental, physiological, or behavioral factors-such as modulating the integrated stress response or improved energy management-may co-evolve with enhanced cognition and contribute to increased lifespan [81,82]. These alternative perspectives warrant exploration in future studies. It should be noted that, although sensory perception controls were not included in this study due to the symmetrical design and focus on relative group differences, we recognize their importance and plan to incorporate them in future studies to minimize any potential confounding effects.

Future research on detailed mechanisms of the genetic linkage between intelligence and longevity would contribute to a biological revolution that leads to a healthier life of humanity. It should be noted that this study using the Drosophila model focused specifically on the relationship between longevity and cognitive ability, addressing a narrow aspect of intelligence rather than intelligence as a comprehensive trait.

## Supporting information

S1 Fig Graphical experimental design of the study. (DOCX)

S2 Fig The lifespan of *D. melanogaster* at each generation during the intelligence selection. a The survival curve of the NINT male and female *D. melanogaster*. b The survival curve of the INT male and female *D. melanogaster.* The lifespan is represented by the Kaplan-Meier survival method.
(DOCX)

S3 Fig The representative H&E-stained brain sections of *D. melanogaster* scoring the degree of neurodegeneration. Histological sections of the brains were scored from 0 to 5 based on the severity of the neurodegeneration by measuring the number of vacuoles as well as their damaged tissue area. Higher score represents a more severe neurodegeneration. Representative images of the H&E-stained brain sections corresponding to each score are as follows: 0, 1 normal to low; 2, 3 moderates; 4, 5 strong to severe.
(DOCX)

S4 Fig The representative H&E-stained histological images of the medullas male and female *D. melanogaster* with age progression. Stained medulla tissues were observed at the magnification of 400X. Scale bar, 50 µm.
(DOCX)

S5 Fig The representative H&E-stained histological images the flight muscles of male and female *D. melanogaster* with age progression. a longitudinal-sections and b cross-sections of the flight muscles of male and female *D. melanogaster* with age progression. Stained flight muscle tissues were observed at the magnification of 100X. Scale bar, 50 µm.
(DOCX)

S6 Fig The startle-induced negative geotaxis assay of male (a) and female (b) *D. melanogaster* with age progression. The startle-induced negative geotaxis assay of male and female *D. melanogaster* with age progression. All data were statistically analyzed by an unpaired Student’s t-test, and values are shown as mean ± SE. *P < 0.05, **P < 0.01 and ***P < 0.001.
(DOCX)

S7 Fig Body weight difference of male and female *D. melanogaster* with age progression. All data were statistically analyzed by an unpaired Student’s t-test, and values are shown as mean ± SE. *P < 0.05 and **P < 0.01.
(DOCX)

S8 Fig The bioanalyzer quality control data. a, c The migration pattern of the total mRNA of male and female *D. melanogaster.* b, d The peak patter of the total mRNA of male and female *D. melanogaster.* (DOCX)

S9 Fig The schematic illustration of total mRNA sequencing. (DOCX)

S10 Fig The principal component analysis (PCA) for RNA-seq sample quality. a-c The principal component analysis (PCA) for RNA-seq sample quality for each comparison group. INT-F_0_: INT compared to F_0_ (a)_,_ NINT-F_0_: NINT compared to F_0_ (b) and INT- NINT: INT compared to NINT (c).
(DOCX)

S1 Table List of the fly strains used to establish F_0_ generation. (DOCX)

S2 Table The composition of *Drosophila* standard cornmeal–agar–molasses medium (for 1 Liter). (DOCX)

S3 Table The quality data of male mRNA used for total mRNA sequencing (RNA extraction quality). (DOCX)

S4 Table The quality data of female mRNA used for total mRNA sequencing (RNA extraction quality). (DOCX)

S5 Table The list of the upregulated genes in each population. (DOCX)

S6 Table The list of the downregulated genes in each population. (DOCX)

S7 Table The list of the Kyoto Encyclopedia of Genes and Genomes (KEGG) pathway. (DOCX)

S8 Table List of GO upregulated. (XLSX)

S9 Table List of GO downregulated. (XLSX)
