# Supplementary figures and images for: Genetic association of intelligence with longevity in Drosophila melanogaster
Source: PLoS One. 2025 Jul 2;20(7):e0325154. doi: 10.1371/journal.pone.0325154 (PMC12221060; doi:10.1371/journal.pone.0325154)

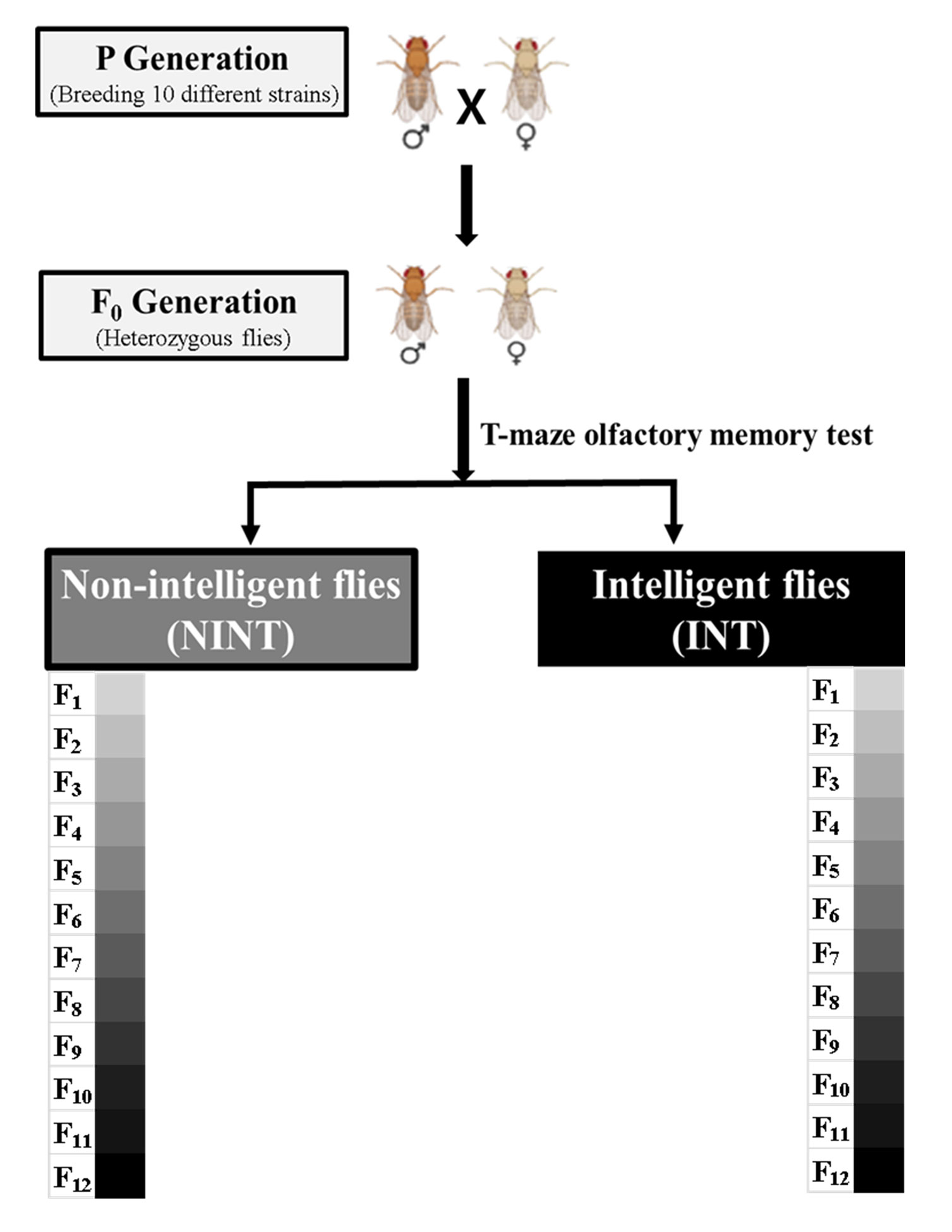


**Supplementary Figure 1. Graphical experimental design of the study.**

Supplement: S1 Fig — (DOCX) [file pone.0325154.s001.docx]

**
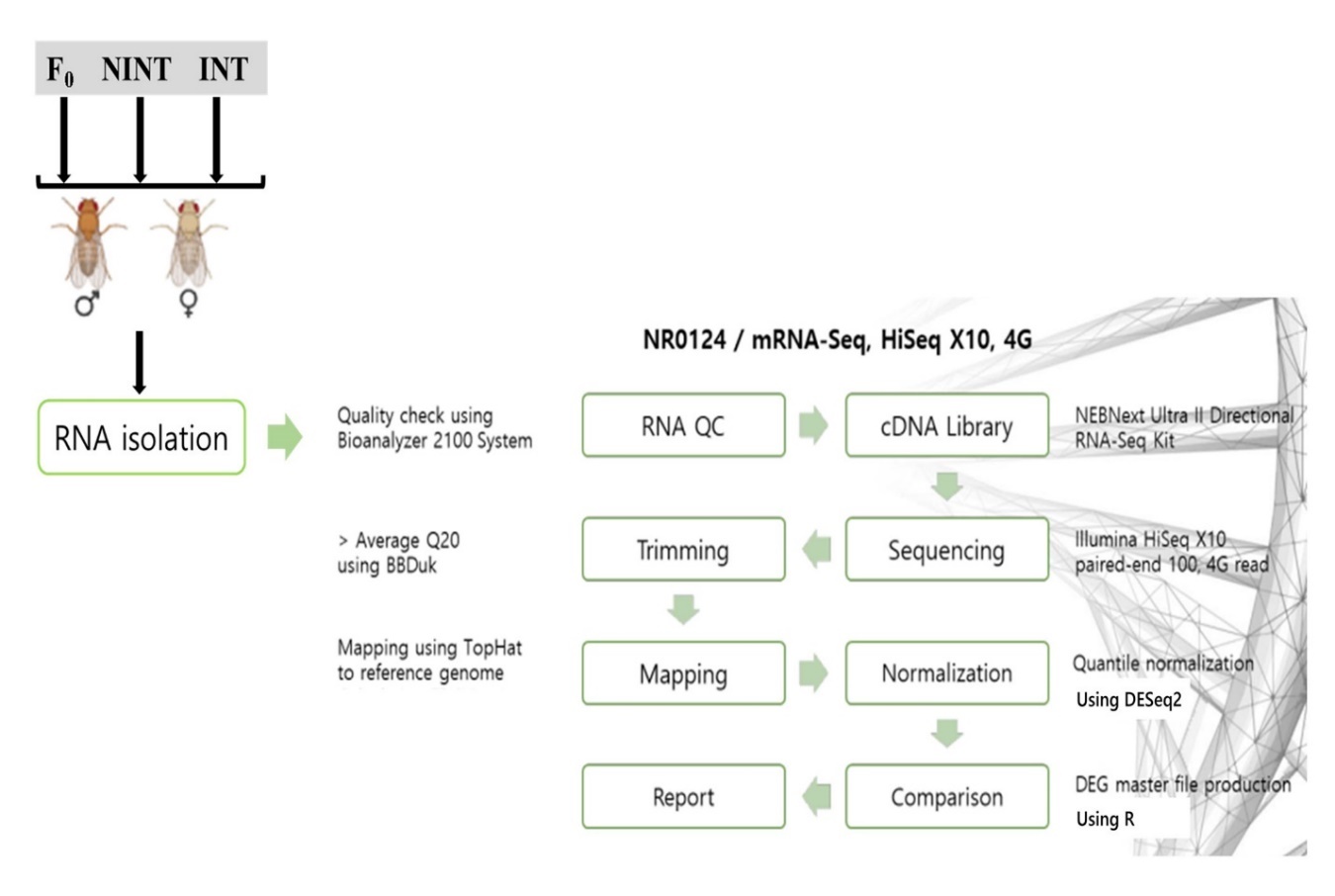
**

**Supplementary Figure 9. The schematic illustration of total mRNA sequencing.**

Supplement: S9 Fig — (DOCX) [file pone.0325154.s009.docx]
