# Supplementary material for: Genetic association of intelligence with longevity in Drosophila melanogaster
Source: PLoS One. 2025 Jul 2;20(7):e0325154. doi: 10.1371/journal.pone.0325154 (PMC12221060; doi:10.1371/journal.pone.0325154)
Supplement: S2 Fig — The lifespan is represented by the Kaplan-Meier survival method. (DOCX) [file pone.0325154.s002.docx]

**
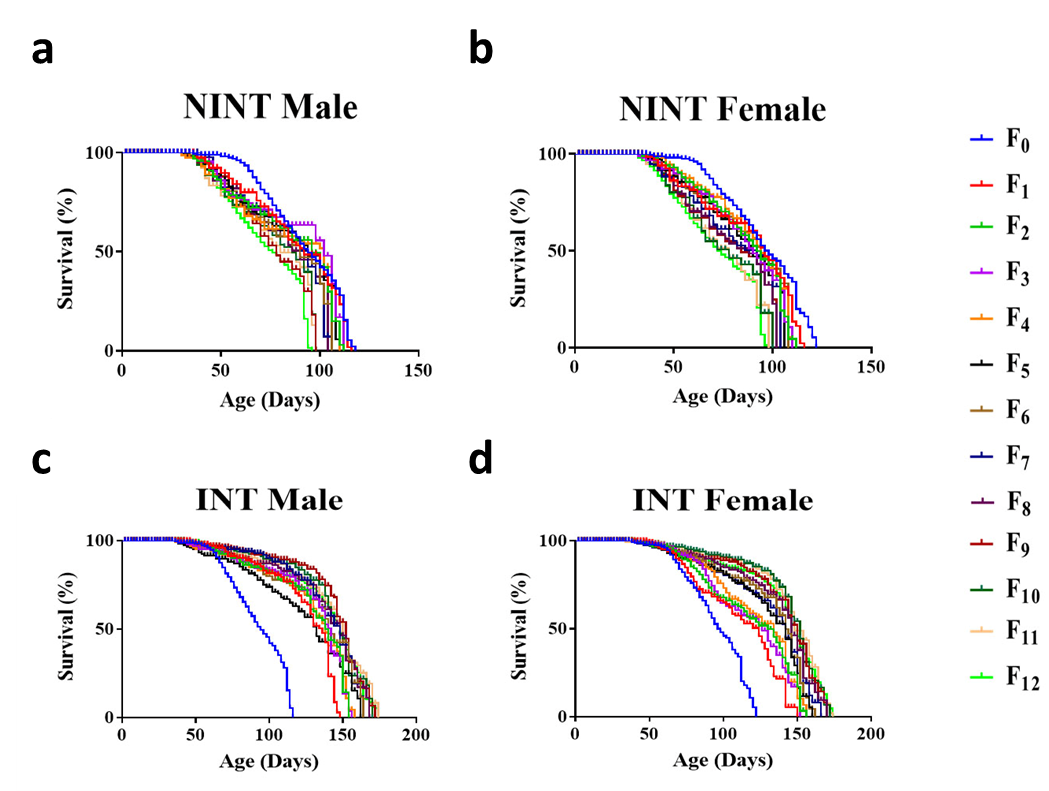
**

**Supplementary Figure 2. The lifespan of *D. melanogaster* at each generation during the intelligence selection. a-b** The survival curve of the NINT male and female *D. melanogaster*. **c-d** The survival curve of the INT male and female *D. melanogaster*. The lifespan is represented by the Kaplan-Meier survival method.
