# Supplementary material for: Genetic association of intelligence with longevity in Drosophila melanogaster
Source: PLoS One. 2025 Jul 2;20(7):e0325154. doi: 10.1371/journal.pone.0325154 (PMC12221060; doi:10.1371/journal.pone.0325154)
Supplement: S3 Fig — Histological sections of the brains were scored from 0 to 5 based on the severity of the neurodegeneration by measuring the number of vacuoles as well as their damaged tissue area. Higher score represents a more severe neurodegeneration. Representative images of the H&E-stained brain sections corresponding to each score are as follows: 0, 1 normal to low; 2, 3 moderates; 4, 5 strong to severe. (DOCX) [file pone.0325154.s003.docx]

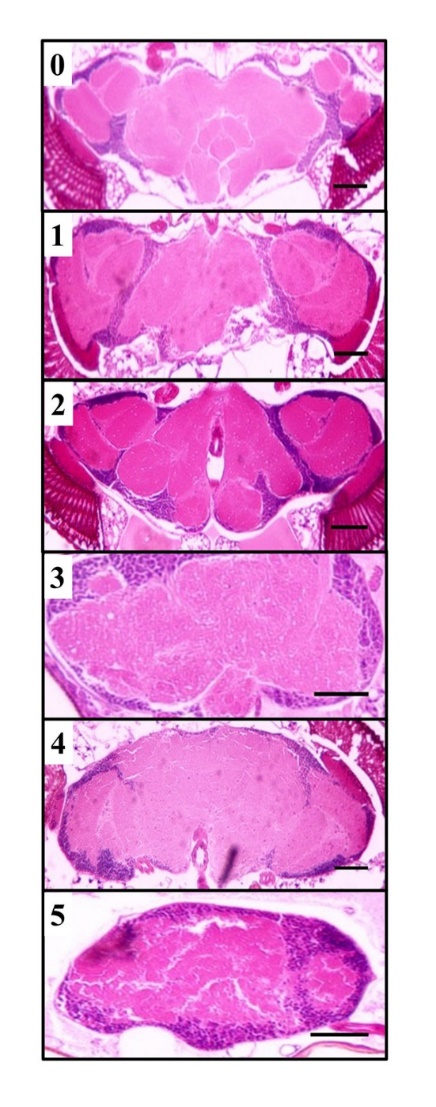


**Supplementary Figure 3. The representative H&E-stained brain sections of *D. melanogaster* scoring the degree of neurodegeneration.** Histological sections of the brains were scored from 0 to 5 based on the severity of the neurodegeneration by measuring the number of vacuoles as well as their damaged tissue area. Higher score represents a more severe neurodegeneration. Representative images of the H&E-stained brain sections corresponding to each score are as follows: 0, 1 normal to low; 2, 3 moderates; 4, 5 strong to severe.
