# Supplementary material for: Genetic association of intelligence with longevity in Drosophila melanogaster
Source: PLoS One. 2025 Jul 2;20(7):e0325154. doi: 10.1371/journal.pone.0325154 (PMC12221060; doi:10.1371/journal.pone.0325154)
Supplement: S4 Fig — Stained medulla tissues were observed at the magnification of 400X. Scale bar, 50 µm. (DOCX) [file pone.0325154.s004.docx]

**
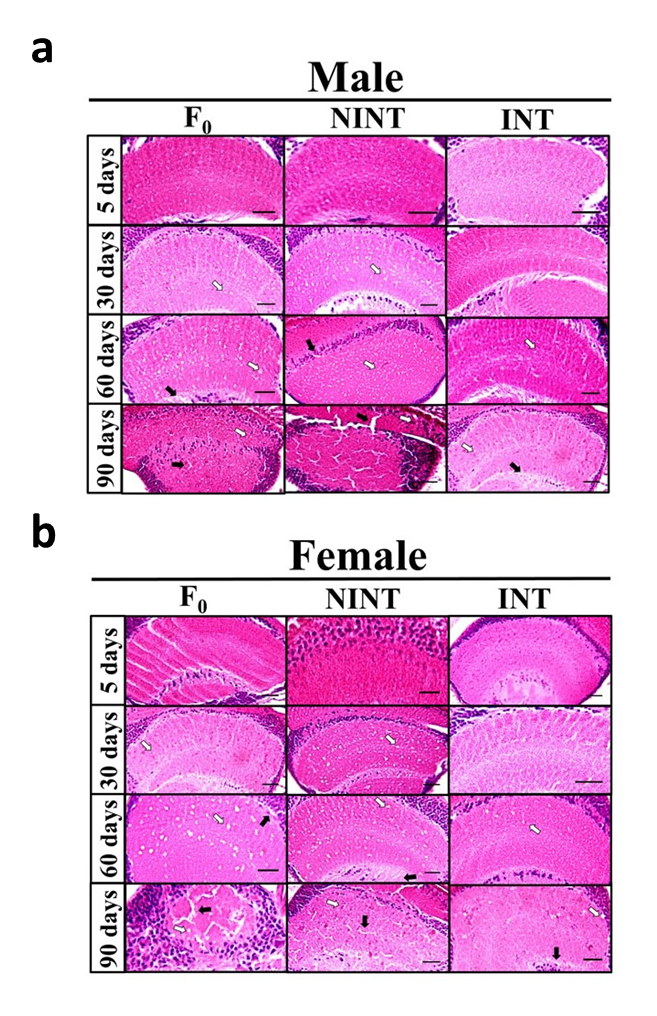
**

**Supplementary Figure 4. The representative H&E-stained histological images of the medullas male (a) and female (b) *D. melanogaster* with age progression.** Stained medulla tissues were observed at the magnification of 400X. Scale bar, 50 µm.
