# Supplementary material for: Genetic association of intelligence with longevity in Drosophila melanogaster
Source: PLoS One. 2025 Jul 2;20(7):e0325154. doi: 10.1371/journal.pone.0325154 (PMC12221060; doi:10.1371/journal.pone.0325154)
Supplement: S5 Fig — Stained flight muscle tissues were observed at the magnification of 100X. Scale bar, 50 µm. (DOCX) [file pone.0325154.s005.docx]

**
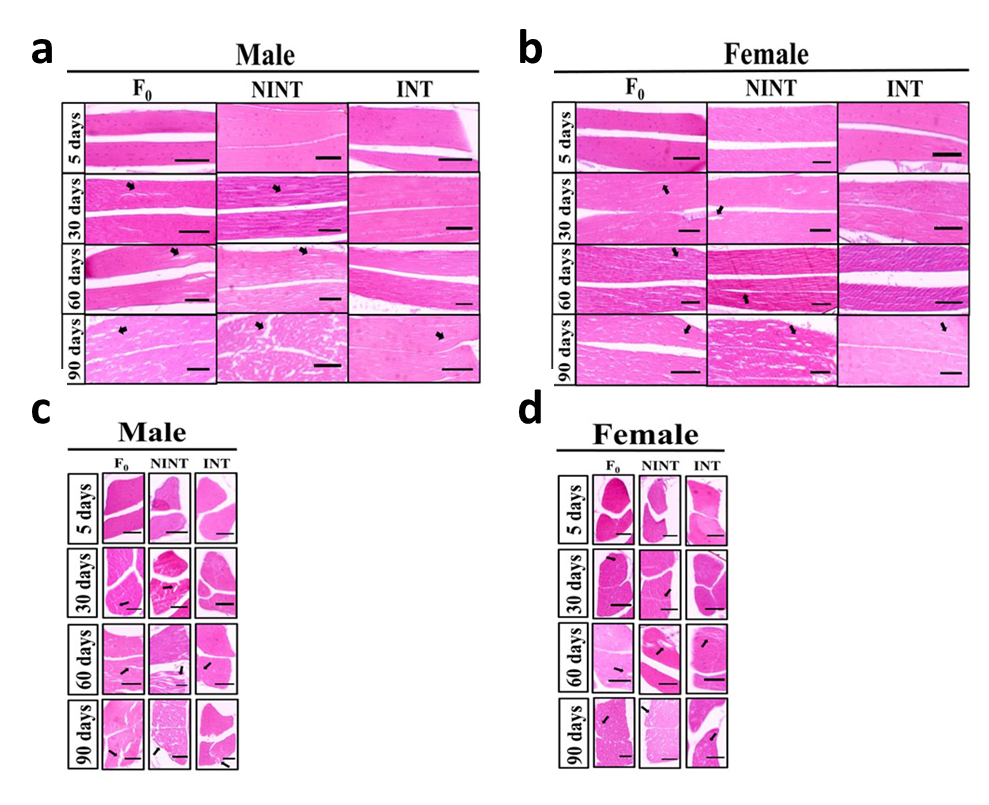
**

**Supplementary Figure 5. The representative H&E-stained histological images the flight muscles of male and female *D. melanogaster* with age progression**. **a-b** longitudinal-sections and **c-d** cross-sections of the flight muscles of male and female *D. melanogaster* with age progression. Stained flight muscle tissues were observed at the magnification of 100X. Scale bar, 50 µm.
