# Supplementary material for: Genetic association of intelligence with longevity in Drosophila melanogaster
Source: PLoS One. 2025 Jul 2;20(7):e0325154. doi: 10.1371/journal.pone.0325154 (PMC12221060; doi:10.1371/journal.pone.0325154)
Supplement: S6 Fig — The startle-induced negative geotaxis assay of male and female D. melanogaster with age progression. All data were statistically analyzed by an unpaired Student’s t-test, and values are shown as mean ± SE. *P < 0.05, **P < 0.01 and ***P < 0.001. (DOCX) [file pone.0325154.s006.docx]

**
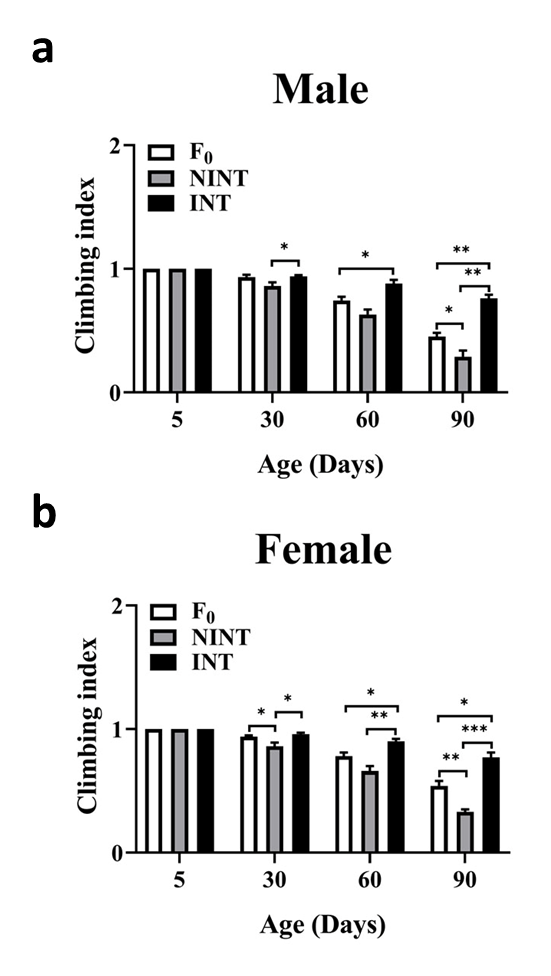
**

**Supplementary Figure 6. The startle-induced negative geotaxis assay of male (a) and female (b) *D. melanogaster* with age progression.** The startle-induced negative geotaxis assay of male and female *D. melanogaster* with age progression. All data were statistically analyzed by an unpaired Student’s t-test, and values are shown as mean ± SE. **P* < 0.05, ***P* < 0.01 and ****P* < 0.001.
