# Supplementary material for: Genetic association of intelligence with longevity in Drosophila melanogaster
Source: PLoS One. 2025 Jul 2;20(7):e0325154. doi: 10.1371/journal.pone.0325154 (PMC12221060; doi:10.1371/journal.pone.0325154)
Supplement: S7 Fig — All data were statistically analyzed by an unpaired Student’s t-test, and values are shown as mean ± SE. *P < 0.05 and **P < 0.01. (DOCX) [file pone.0325154.s007.docx]

**
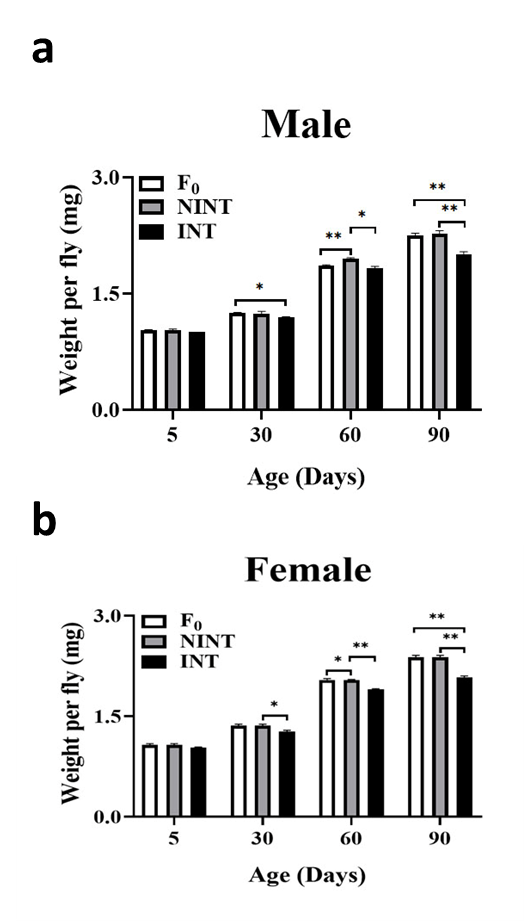
**

**S7 Fig.** **Body weight difference of male (a) and female (b) *D. melanogaster* with age progression.** All data were statistically analyzed by an unpaired Student’s t-test, and values are shown as mean ± SE. **P* < 0.05 and ***P* < 0.01.Supplementary Figure 8. The bioanalyzer quality control data.
