# Supplementary material for: Genetic association of intelligence with longevity in Drosophila melanogaster
Source: PLoS One. 2025 Jul 2;20(7):e0325154. doi: 10.1371/journal.pone.0325154 (PMC12221060; doi:10.1371/journal.pone.0325154)
Supplement: S8 Fig — (DOCX) [file pone.0325154.s008.docx]

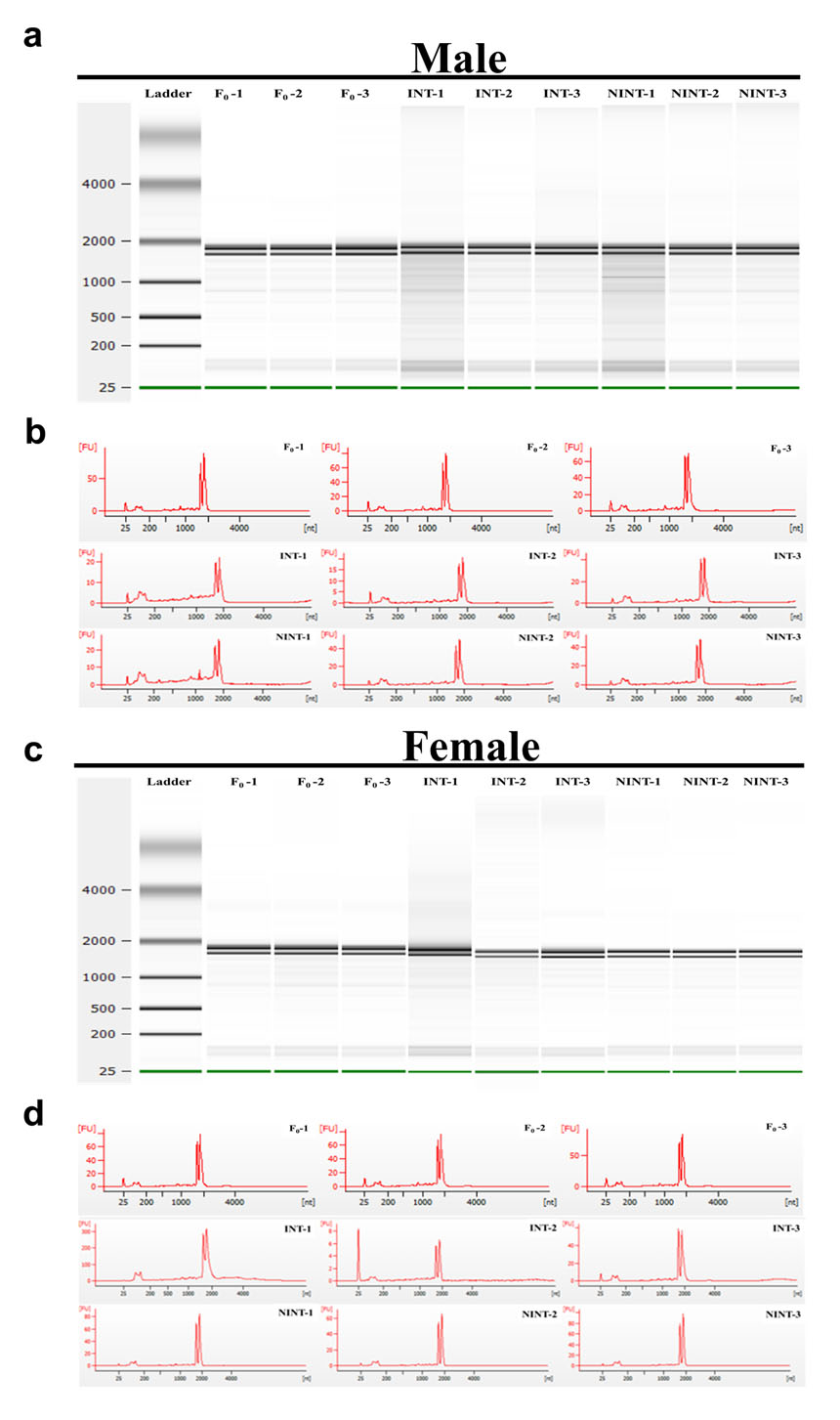


**S8 Fig.** **The bioanalyzer quality control data. a**, **c** The migration pattern of the total mRNA of male and female *D. melanogaster.* **b**, **d** The peak patter of the total mRNA of male and female *D. melanogaster.*
