# Supplementary material for: Genetic association of intelligence with longevity in Drosophila melanogaster
Source: PLoS One. 2025 Jul 2;20(7):e0325154. doi: 10.1371/journal.pone.0325154 (PMC12221060; doi:10.1371/journal.pone.0325154)
Supplement: S10 Fig — INT-F0: INT compared to F0 (a), NINT-F0: NINT compared to F0 (b) and INT- NINT: INT compared to NINT (c). (DOCX) [file pone.0325154.s010.docx]

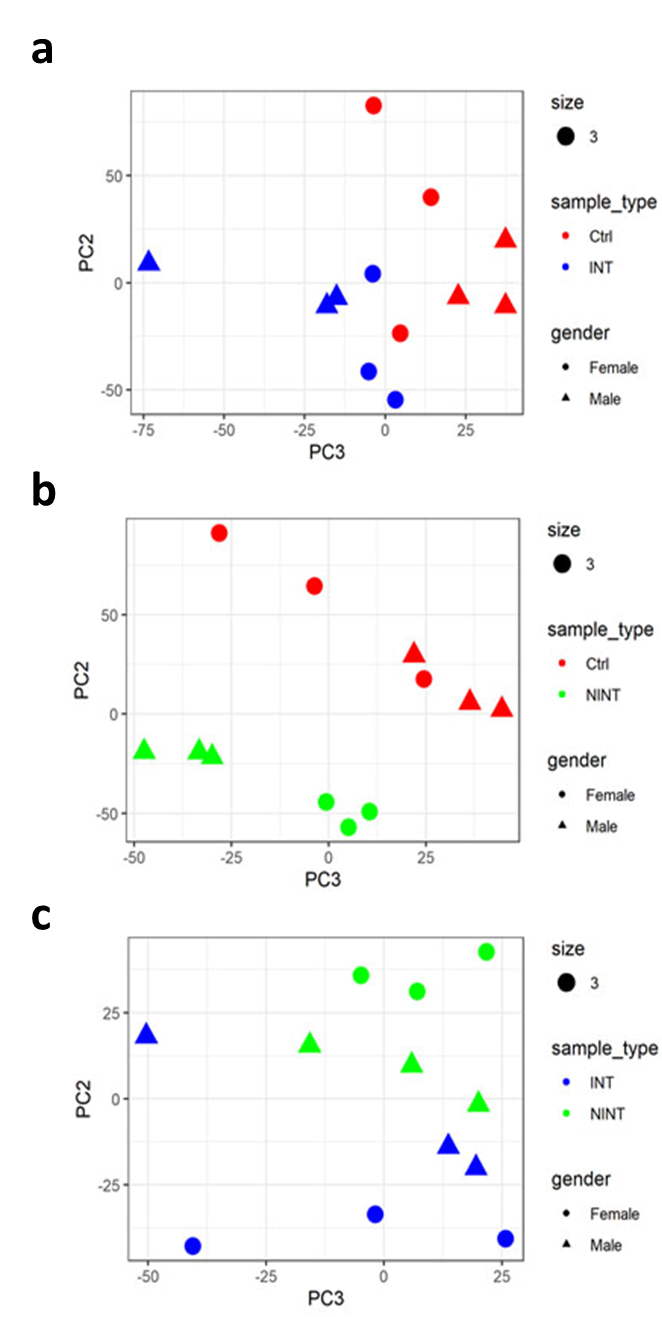


**Supplementary Figure 10. The principal component analysis (PCA) for RNA-seq sample quality. a-c** The principal component analysis (PCA) for RNA-seq sample quality for each comparison group. INT-F_0_: INT compared to F_0_ (**a**)_,_ NINT-F_0_: NINT compared to F_0_ (**b**) and INT- NINT: INT compared to NINT (**c**).
