# Supplementary material for: Genetic association of intelligence with longevity in Drosophila melanogaster
Source: PLoS One. 2025 Jul 2;20(7):e0325154. doi: 10.1371/journal.pone.0325154 (PMC12221060; doi:10.1371/journal.pone.0325154)
Supplement: S1 Table — (DOCX) [file pone.0325154.s011.docx]

**Supplementary Table 1. List of the fly strains used to establish F_0_ generation**

| **Strain** | **Genotype** | **Source** |
| --- | --- | --- |
| Oregon-R-C | [Oregon-R-C](http://flybase.org/reports/FBsn0000277.html) | BDSC# [5](http://flystocks.bio.indiana.edu/Reports/5.html) |
| DGRP-100 | [DGRP-100](http://flybase.org/reports/FBsn0000288.html) | BDSC# [55017](http://flystocks.bio.indiana.edu/Reports/55017.html) |
| BER_2 | [BER_2](http://flybase.org/reports/FBsn0000223.html) | BDSC# [3840](http://flystocks.bio.indiana.edu/Reports/3840.html) |
| Harwich | [Harwich](http://flybase.org/reports/FBsn0000304.html) | BDSC# [4264](http://flystocks.bio.indiana.edu/Reports/4264.html) |
| Canton-S | [Canton-S](http://flybase.org/reports/FBsn0000274.html) | BDSC# [64349](http://flystocks.bio.indiana.edu/Reports/64349.html) |
| Wild_1A | [Wild_1A](http://flybase.org/reports/FBsn0000261.html) | BDSC# [3878](http://flystocks.bio.indiana.edu/Reports/3878.html) |
| w[1118] | [w^1118^](http://flybase.org/reports/FBal0018186.html) | BDSC# [5905](http://bdsc.indiana.edu/stocks/5905) |
