# Supplementary material for: Genetic association of intelligence with longevity in Drosophila melanogaster
Source: PLoS One. 2025 Jul 2;20(7):e0325154. doi: 10.1371/journal.pone.0325154 (PMC12221060; doi:10.1371/journal.pone.0325154)
Supplement: S2 Table — (DOCX) [file pone.0325154.s012.docx]

**Supplementary Table 2.** **The composition of *Drosophila* standard cornmeal–agar–molasses medium (for 1 Liter)**

| **Ingredients** | **Amount** |
| --- | --- |
| Corn meal | 84 gm |
| Yeast | 24 gm |
| Sucrose | 47 gm |
| Agar* | 6/8 gm |
| Distilled water | 1 liter |
| Molasses | 25 ml |
| 10% methyl perahydroxybenzoate | 10 ml |
| Propionic acid | 4 ml |

* Agar is needed in the amount of 6gm and 8 gm in soft and hard medium, respectively.
