# Supplementary material for: Genetic association of intelligence with longevity in Drosophila melanogaster
Source: PLoS One. 2025 Jul 2;20(7):e0325154. doi: 10.1371/journal.pone.0325154 (PMC12221060; doi:10.1371/journal.pone.0325154)
Supplement: S3 Table — (DOCX) [file pone.0325154.s013.docx]

**Supplementary Table 3. The quality data of male mRNA used for total mRNA sequencing (RNA extraction quality)**

| **Sample** | **Fly numbers** | **Age (Days)** | **Conc.**  **(ng µl^-1^)** | **Total (µg)** | **OD_260/280_** | **OD_260/230_** | **RIN** | **Result** |
| --- | --- | --- | --- | --- | --- | --- | --- | --- |
| F_0_-1 | 100 | 5 | 1934.4 | 218.587 | 2.15 | 1.58 | 6.3 | Pass |
| F_0_-2 | 100 | 5 | 2036.4 | 228.081 | 2.14 | 1.35 | 6.3 | Pass |
| F_0_-3 | 100 | 5 | 1538.2 | 169.199 | 2.17 | 1.76 | 6.4 | Pass |
| INT-1 | 100 | 5 | 3261.0 | 101.090 | 2.03 | 1.99 | 5.1 | Pass |
| INT-2 | 100 | 5 | 2958.8 | 91.722 | 2.10 | 2.05 | 6.1 | Pass |
| INT-3 | 100 | 5 | 2841.7 | 88.092 | 2.08 | 2.05 | 5.9 | Pass |
| NINT-1 | 100 | 5 | 3489.8 | 108.183 | 2.01 | 2.24 | 4.9 | Pass |
| NINT-2 | 100 | 5 | 3573.3 | 110.774 | 1.98 | 2.03 | 6.1 | Pass |
| NINT-3 | 100 | 5 | 3488.3 | 108.137 | 2.01 | 2.05 | 6.0 | Pass |
