# Supplementary material for: Genetic association of intelligence with longevity in Drosophila melanogaster
Source: PLoS One. 2025 Jul 2;20(7):e0325154. doi: 10.1371/journal.pone.0325154 (PMC12221060; doi:10.1371/journal.pone.0325154)
Supplement: S4 Table — (DOCX) [file pone.0325154.s014.docx]

**Supplementary Table 4. The quality data of female mRNA used for total mRNA sequencing (RNA extraction quality)**

| **Sample** | **Fly numbers** | **Age (Days)** | **Conc.**  **(ng µl^-1^)** | **Total (µg)** | **OD_260/280_** | **OD_260/230_** | **RIN** | **Result** |
| --- | --- | --- | --- | --- | --- | --- | --- | --- |
| F_0_-1 | 100 | 5 | 3994.9 | 439.437 | 1.58 | 1.32 | 6.5 | Pass |
| F_0_-2 | 100 | 5 | 3631.7 | 406.754 | 1.95 | 1.40 | 6.3 | Pass |
| F_0_-3 | 100 | 5 | 3752.1 | 423.985 | 1.90 | 1.34 | 6.4 | Pass |
| INT-1 | 100 | 5 | 3018.3 | 87.53 | 2.02 | 2.20 | 6.5 | Pass |
| INT-2 | 100 | 5 | 4108.1 | 119.13 | 1.89 | 2.01 | 6.9 | Pass |
| INT-3 | 100 | 5 | 3756.1 | 108.93 | 1.94 | 2.06 | 7.3 | Pass |
| NINT-1 | 100 | 5 | 3833.6 | 191.68 | 1.89 | 2.01 | 6.3 | Pass |
| NINT-2 | 100 | 5 | 3768.4 | 188.42 | 1.90 | 2.01 | 6.3 | Pass |
| NINT-3 | 100 | 5 | 4238.4 | 211.92 | 1.93 | 2.05 | 6.3 | Pass |
