# Supplementary material for: Genetic association of intelligence with longevity in Drosophila melanogaster
Source: PLoS One. 2025 Jul 2;20(7):e0325154. doi: 10.1371/journal.pone.0325154 (PMC12221060; doi:10.1371/journal.pone.0325154)
Supplement: S5 Table — (DOCX) [file pone.0325154.s015.docx]

**Supplementary Table 5. The list of the upregulated genes in each population**

| **INT (compared with F_0_)** | | | | | | | |
| --- | --- | --- | --- | --- | --- | --- | --- |
|  | **Gene symbol** | **baseMean** | **log2FoldChange** | **lfcSE** | **stat** | ***P*value** | ***P*adj** |
| 56 | Acp24A4 | 2951.112 | 11.720 | 1.821 | 6.436 | 1.23E-10 | 1.44E-07 |
| 55 | Ser12 | 78.197 | 9.029 | 1.855 | 4.867 | 1.13E-06 | 4.04E-04 |
| 54 | CR31084 | 334.968 | 7.543 | 0.799 | 9.445 | 3.54E-21 | 2.90E-17 |
| 53 | CG42876 | 40.805 | 7.046 | 1.215 | 5.800 | 6.62E-09 | 4.72281E-06 |
| 52 | CR45530 | 2689.245 | 6.305 | 1.327 | 4.752 | 2.01E-06 | 6.87E-04 |
| 51 | CR44463 | 9.070 | 5.998 | 1.308 | 4.584 | 4.55E-06 | 0.001287735 |
| 50 | CG7542 | 1517.173 | 4.425 | 0.581 | 7.616 | 2.61E-14 | 5.36E-11 |
| 49 | CG4927 | 66.973 | 4.233 | 0.809 | 5.235 | 1.65E-07 | 7.95E-05 |
| 48 | CG43165 | 13.065 | 4.191 | 1.003 | 4.178 | 2.94E-05 | 6.04E-03 |
| 47 | CR43452 | 10.325 | 4.130 | 0.832 | 4.964 | 6.89E-07 | 2.76E-04 |
| 46 | Cyp6a8 | 146.870 | 4.003 | 0.640 | 6.251 | 4.08E-10 | 4.1789E-07 |
| 45 | CG4757 | 692.579 | 3.598 | 0.661 | 5.441 | 5.30E-08 | 2.81E-05 |
| 44 | CG34291 | 532.532 | 3.588 | 0.845 | 4.247 | 2.17E-05 | 4.77E-03 |
| 43 | CG10725 | 432.713 | 3.344 | 0.541 | 6.177 | 6.54E-10 | 6.26E-07 |
| 42 | CG11034 | 992.056 | 3.308 | 0.313 | 10.584 | 3.55E-26 | 5.82E-22 |
| 41 | CG2772 | 209.525 | 3.291 | 0.536 | 6.140 | 8.27E-10 | 7.14E-07 |
| 40 | CG11893 | 253.204 | 3.018 | 0.473 | 6.376 | 1.82E-10 | 1.99E-07 |
| 39 | CG10140 | 12.225 | 2.923 | 0.669 | 4.367 | 1.26E-05 | 2.99E-03 |
| 38 | CG13324 | 385.953 | 2.753 | 0.551 | 4.996 | 5.85E-07 | 2.46E-04 |
| 37 | CG8539 | 238.201 | 2.680 | 0.305 | 8.782 | 1.61E-18 | 6.60E-15 |
| 36 | CG11700 | 2357.666 | 2.679 | 0.538 | 4.976 | 6.49E-07 | 2.66E-04 |
| 35 | CR43651 | 133.288 | 2.480 | 0.335 | 7.408 | 1.29E-13 | 2.11E-10 |
| 34 | CG13454 | 9.840 | 2.464 | 0.529 | 4.659 | 3.18E-06 | 9.48E-04 |
| 33 | CG33337 | 15.856 | 2.244 | 0.497 | 4.515 | 6.33E-06 | 1.73E-03 |
| 32 | CG16704 | 421.585 | 2.122 | 0.435 | 4.876 | 1.08E-06 | 3.94E-04 |
| 31 | CG42329 | 143.440 | 1.799 | 0.371 | 4.854 | 1.21068E-06 | 0.000422604 |
| 30 | CG12204 | 445.390 | 1.766 | 0.257 | 6.864 | 6.72E-12 | 8.47509E-09 |
| 29 | CR42861 | 164.493 | 1.657 | 0.350 | 4.730 | 2.25E-06 | 0.000723346 |
| 28 | CG33774 | 386.998 | 1.579 | 0.259 | 6.087 | 1.15E-09 | 9.42574E-07 |
| 27 | CG32641 | 1415.952 | 1.528 | 0.190 | 8.052 | 8.14E-16 | 2.65636E-12 |
| 26 | CR44030 | 235.266 | 1.416 | 0.315 | 4.498 | 6.86E-06 | 0.001844422 |
| 25 | CG14120 | 1598.932 | 1.355 | 0.329 | 4.114 | 3.90E-05 | 0.007890338 |
| 24 | CG8768 | 301.080 | 1.355 | 0.147 | 9.243 | 2.39E-20 | 1.30508E-16 |
| 23 | CG14820 | 517.789 | 1.266 | 0.300 | 4.223 | 2.42E-05 | 0.005213584 |
| 22 | GstD3 | 184.374 | 1.240 | 0.305 | 4.065 | 4.81E-05 | 9.29E-03 |
| 21 | Thor | 6842.719 | 1.176 | 0.287 | 4.092 | 4.28E-05 | 0.008459205 |
| 20 | fuss | 265.884 | 1.129 | 0.207 | 5.459 | 4.79E-08 | 2.62E-05 |
| 19 | CG32581 | 2402.815 | 1.079 | 0.182 | 5.942 | 2.82E-09 | 2.10391E-06 |
| 18 | CG32640 | 1384.636 | 1.013 | 0.164 | 6.169 | 6.87E-10 | 6.26371E-07 |
| 17 | snoRNA:Or-CD11a | 4784.590 | 0.978 | 0.190 | 5.147 | 2.65E-07 | 0.0001241 |
| 16 | snoRNA:Or-CD11c | 13618.260 | 0.976 | 0.199 | 4.906 | 9.31E-07 | 0.000347044 |
| 15 | snoRNA:Or-CD11b | 13618.272 | 0.976 | 0.199 | 4.906 | 9.30E-07 | 0.000347044 |
| 14 | CG41378 | 498.083 | 0.966 | 0.216 | 4.481 | 7.44E-06 | 0.001968984 |
| 13 | CG6852 | 1519.523 | 0.956 | 0.132 | 7.237 | 4.58E-13 | 6.83E-10 |
| 12 | snoRNA:Psi28S-1232 | 1236.101 | 0.949 | 0.173 | 5.495 | 3.91E-08 | 2.21352E-05 |
| 11 | CG18417 | 283.237 | 0.922 | 0.165 | 5.577 | 2.45E-08 | 1.55E-05 |
| 10 | CG6094 | 436.089 | 0.841 | 0.200 | 4.197 | 2.70E-05 | 5.63E-03 |
| 9 | CG7054 | 933.555 | 0.833 | 0.198 | 4.201 | 2.65E-05 | 0.005633635 |
| 8 | CG9577 | 1129.868 | 0.769 | 0.103 | 7.451 | 9.24E-14 | 1.68524E-10 |
| 7 | snoRNA:Psi18S-920 | 6624.396 | 0.675 | 0.146 | 4.616 | 3.91E-06 | 0.001144926 |
| 6 | Bet5 | 277.068 | 0.536 | 0.131 | 4.096 | 4.20E-05 | 0.008394285 |
| 5 | CG15432 | 553.003 | 0.506 | 0.120 | 4.196 | 2.71E-05 | 0.005633635 |
| 4 | CG7456 | 1107.933 | 0.491 | 0.104 | 4.712 | 2.46E-06 | 7.71E-04 |
| 3 | GM130 | 1130.337 | 0.432 | 0.078 | 5.523 | 3.34E-08 | 1.96E-05 |
| 2 | CG30118 | 5658.622 | 0.411 | 0.074 | 5.542 | 2.99E-08 | 1.82E-05 |
| 1 | P5cr | 465.193 | 0.381 | 0.090 | 4.246 | 2.18E-05 | 0.004768501 |
| **NINT (compared with F_0_)** | | | | | | | |
|  | **Gene symbol** | **baseMean** | **log2FoldChange** | **lfcSE** | **stat** | ***P*value** | ***P*adj** |
| 152 | Acp24A4 | 2951.112 | 10.022 | 1.821 | 5.504 | 3.719E-08 | 6.780E-06 |
| 151 | Ser12 | 78.197 | 9.388 | 1.855 | 5.062 | 4.158E-07 | 5.016E-05 |
| 150 | CG42876 | 40.805 | 8.291 | 1.212 | 6.842 | 7.783E-12 | 5.804E-09 |
| 149 | Obp47b | 149.550 | 8.013 | 1.615 | 4.962 | 6.961E-07 | 7.465E-05 |
| 148 | CR31084 | 334.968 | 7.942 | 0.798 | 9.949 | 2.554E-23 | 4.191E-19 |
| 147 | Or47b | 301.666 | 7.585 | 1.517 | 5.000 | 5.746E-07 | 6.434E-05 |
| 146 | sha | 1929.002 | 7.302 | 1.326 | 5.509 | 3.608E-08 | 6.650E-06 |
| 145 | Cpr5C | 10.272 | 6.514 | 1.778 | 3.664 | 2.481E-04 | 7.934E-03 |
| 144 | CR44463 | 9.070 | 6.221 | 1.305 | 4.769 | 1.852E-06 | 1.726E-04 |
| 143 | CG4757 | 692.579 | 5.261 | 0.661 | 7.962 | 1.688E-15 | 2.518E-12 |
| 142 | nompA | 1136.894 | 5.123 | 1.014 | 5.055 | 4.302E-07 | 5.152E-05 |
| 141 | CG30098 | 15.838 | 5.052 | 1.091 | 4.632 | 3.616E-06 | 2.951E-04 |
| 140 | CG42729 | 4.833 | 5.001 | 1.027 | 4.868 | 1.128E-06 | 1.102E-04 |
| 139 | CG43165 | 13.065 | 4.667 | 0.998 | 4.677 | 2.905E-06 | 2.508E-04 |
| 138 | CG13324 | 385.953 | 4.667 | 0.550 | 8.488 | 2.099E-17 | 8.607E-14 |
| 137 | CG4927 | 66.973 | 4.596 | 0.807 | 5.692 | 1.258E-08 | 2.680E-06 |
| 136 | CG42728 | 5.022 | 4.503 | 0.967 | 4.659 | 3.184E-06 | 2.671E-04 |
| 135 | CG11313 | 143.923 | 4.137 | 0.897 | 4.615 | 3.932E-06 | 3.162E-04 |
| 134 | CR44371 | 9.798 | 4.106 | 1.048 | 3.919 | 8.904E-05 | 3.652E-03 |
| 133 | CG7542 | 1517.173 | 4.104 | 0.581 | 7.064 | 1.617E-12 | 1.474E-09 |
| 132 | Desat2 | 125.359 | 3.955 | 0.856 | 4.618 | 3.877E-06 | 3.133E-04 |
| 131 | CG34291 | 532.532 | 3.941 | 0.845 | 4.666 | 3.064E-06 | 2.618E-04 |
| 130 | CG10725 | 432.713 | 3.878 | 0.541 | 7.169 | 7.565E-13 | 7.757E-10 |
| 129 | CG13646 | 12.220 | 3.836 | 0.916 | 4.187 | 2.829E-05 | 1.532E-03 |
| 128 | Cyp6a8 | 146.870 | 3.813 | 0.640 | 5.957 | 2.577E-09 | 7.046E-07 |
| 127 | CR44640 | 18.033 | 3.601 | 0.890 | 4.048 | 5.166E-05 | 2.354E-03 |
| 126 | CG12708 | 103.151 | 3.597 | 0.771 | 4.668 | 3.035E-06 | 2.607E-04 |
| 125 | CG14329 | 16.504 | 3.581 | 0.882 | 4.060 | 4.914E-05 | 2.284E-03 |
| 124 | CG7741 | 2210.587 | 3.574 | 0.762 | 4.693 | 2.696E-06 | 2.365E-04 |
| 123 | TotM | 149.558 | 3.538 | 0.606 | 5.843 | 5.114E-09 | 1.234E-06 |
| 122 | CG10140 | 12.225 | 3.483 | 0.660 | 5.279 | 1.300E-07 | 1.918E-05 |
| 121 | Cda9 | 308.702 | 3.459 | 0.761 | 4.544 | 5.522E-06 | 4.137E-04 |
| 120 | Ir40a | 58.784 | 3.410 | 0.701 | 4.868 | 1.126E-06 | 1.102E-04 |
| 119 | CG13905 | 680.004 | 3.347 | 0.597 | 5.607 | 2.053E-08 | 4.066E-06 |
| 118 | CG17191 | 20.598 | 3.227 | 0.819 | 3.939 | 8.181E-05 | 3.372E-03 |
| 117 | CR43857 | 38.537 | 3.120 | 0.695 | 4.493 | 7.028E-06 | 5.057E-04 |
| 116 | Sox21a | 54.647 | 3.109 | 0.747 | 4.161 | 3.172E-05 | 1.673E-03 |
| 115 | CG11893 | 253.204 | 3.096 | 0.473 | 6.544 | 5.971E-11 | 2.721E-08 |
| 114 | CG33137 | 13.435 | 3.092 | 0.838 | 3.690 | 2.240E-04 | 7.365E-03 |
| 113 | CG30033 | 411.798 | 3.090 | 0.715 | 4.324 | 1.531E-05 | 9.586E-04 |
| 112 | CG2772 | 209.525 | 3.053 | 0.536 | 5.696 | 1.227E-08 | 2.648E-06 |
| 111 | CG11034 | 992.056 | 2.961 | 0.313 | 9.472 | 2.740E-21 | 2.248E-17 |
| 110 | CR43651 | 133.288 | 2.952 | 0.333 | 8.861 | 7.956E-19 | 4.351E-15 |
| 109 | CG12970 | 28.386 | 2.913 | 0.696 | 4.187 | 2.826E-05 | 1.532E-03 |
| 108 | CG32302 | 109.913 | 2.864 | 0.549 | 5.214 | 1.846E-07 | 2.503E-05 |
| 107 | CG16704 | 421.585 | 2.812 | 0.435 | 6.470 | 9.807E-11 | 4.234E-08 |
| 106 | CG9759 | 593.736 | 2.677 | 0.694 | 3.860 | 1.134E-04 | 4.440E-03 |
| 105 | CG13461 | 29.497 | 2.620 | 0.715 | 3.662 | 2.500E-04 | 7.965E-03 |
| 104 | CG8539 | 238.201 | 2.493 | 0.305 | 8.173 | 3.007E-16 | 6.167E-13 |
| 103 | CG43773 | 51.666 | 2.489 | 0.425 | 5.861 | 4.612E-09 | 1.146E-06 |
| 102 | CG33337 | 15.856 | 2.456 | 0.491 | 5.004 | 5.624E-07 | 6.363E-05 |
| 101 | CG43187 | 29.348 | 2.425 | 0.495 | 4.899 | 9.621E-07 | 9.743E-05 |
| 100 | CR42646 | 57.872 | 2.383 | 0.662 | 3.599 | 3.194E-04 | 9.614E-03 |
| 99 | CG32284 | 144.802 | 2.362 | 0.599 | 3.941 | 8.110E-05 | 3.360E-03 |
| 98 | CR45045 | 188.460 | 2.348 | 0.607 | 3.866 | 1.107E-04 | 4.376E-03 |
| 97 | Osi8 | 26.236 | 2.092 | 0.550 | 3.804 | 1.423E-04 | 5.245E-03 |
| 96 | CR42874 | 9.002 | 2.022 | 0.531 | 3.804 | 1.421E-04 | 5.245E-03 |
| 95 | CG12520 | 32.026 | 1.991 | 0.441 | 4.512 | 6.411E-06 | 4.695E-04 |
| 94 | Sox100B | 80.542 | 1.913 | 0.475 | 4.025 | 5.703E-05 | 2.515E-03 |
| 93 | Npc2e | 548.008 | 1.868 | 0.479 | 3.902 | 9.531E-05 | 3.871E-03 |
| 92 | Damm | 1115.414 | 1.853 | 0.493 | 3.759 | 1.708E-04 | 5.944E-03 |
| 91 | CG3259 | 64.863 | 1.808 | 0.456 | 3.966 | 7.305E-05 | 3.081E-03 |
| 90 | CG4259 | 314.122 | 1.764 | 0.395 | 4.465 | 7.991E-06 | 5.607E-04 |
| 89 | CR42861 | 164.493 | 1.760 | 0.350 | 5.033 | 4.826E-07 | 5.656E-05 |
| 88 | CG11261 | 261.255 | 1.712 | 0.315 | 5.431 | 5.603E-08 | 9.677E-06 |
| 87 | CG12204 | 445.390 | 1.708 | 0.257 | 6.647 | 2.995E-11 | 1.820E-08 |
| 86 | CG42329 | 143.440 | 1.621 | 0.370 | 4.377 | 1.205E-05 | 7.939E-04 |
| 85 | GstD3 | 184.374 | 1.571 | 0.304 | 5.169 | 2.354E-07 | 3.090E-05 |
| 84 | CR44192 | 618.494 | 1.535 | 0.341 | 4.503 | 6.689E-06 | 4.855E-04 |
| 83 | CG32641 | 1415.952 | 1.508 | 0.190 | 7.952 | 1.842E-15 | 2.519E-12 |
| 82 | CR44030 | 235.266 | 1.501 | 0.314 | 4.776 | 1.784E-06 | 1.672E-04 |
| 81 | CG1529 | 893.262 | 1.498 | 0.364 | 4.117 | 3.839E-05 | 1.903E-03 |
| 80 | CG33774 | 386.998 | 1.471 | 0.259 | 5.679 | 1.354E-08 | 2.848E-06 |
| 79 | CG13902 | 297.886 | 1.411 | 0.318 | 4.430 | 9.442E-06 | 6.454E-04 |
| 78 | CG11842 | 398.640 | 1.396 | 0.383 | 3.640 | 2.729E-04 | 8.529E-03 |
| 77 | fuss | 265.884 | 1.326 | 0.206 | 6.442 | 1.180E-10 | 4.609E-08 |
| 76 | Cht8 | 1073.713 | 1.253 | 0.227 | 5.526 | 3.278E-08 | 6.182E-06 |
| 75 | snoRNA:Me28S-C788b | 84.199 | 1.135 | 0.232 | 4.895 | 9.828E-07 | 9.842E-05 |
| 74 | CG11695 | 518.250 | 1.117 | 0.311 | 3.593 | 3.264E-04 | 9.772E-03 |
| 73 | l(2)not | 573.352 | 1.111 | 0.290 | 3.829 | 1.288E-04 | 4.901E-03 |
| 72 | CR45341 | 46.993 | 1.101 | 0.268 | 4.104 | 4.064E-05 | 1.977E-03 |
| 71 | dgo | 323.496 | 1.069 | 0.232 | 4.605 | 4.123E-06 | 3.236E-04 |
| 70 | gny | 234.429 | 1.067 | 0.178 | 6.002 | 1.950E-09 | 5.714E-07 |
| 69 | Thor | 6842.719 | 1.048 | 0.287 | 3.647 | 2.653E-04 | 8.370E-03 |
| 68 | CG8768 | 301.080 | 1.043 | 0.146 | 7.122 | 1.061E-12 | 1.024E-09 |
| 67 | CG5773 | 1754.914 | 1.025 | 0.247 | 4.144 | 3.418E-05 | 1.741E-03 |
| 66 | CG14906 | 215.015 | 1.018 | 0.233 | 4.372 | 1.232E-05 | 8.086E-04 |
| 65 | CG32581 | 2402.815 | 1.018 | 0.182 | 5.607 | 2.057E-08 | 4.066E-06 |
| 64 | CG13102 | 518.128 | 1.015 | 0.222 | 4.565 | 4.994E-06 | 3.811E-04 |
| 63 | CG4496 | 249.846 | 0.974 | 0.251 | 3.880 | 1.046E-04 | 4.153E-03 |
| 62 | CG31075 | 1553.636 | 0.974 | 0.226 | 4.308 | 1.648E-05 | 1.012E-03 |
| 61 | CG6852 | 1519.523 | 0.959 | 0.132 | 7.271 | 3.556E-13 | 3.889E-10 |
| 60 | CG6928 | 840.026 | 0.959 | 0.231 | 4.152 | 3.295E-05 | 1.705E-03 |
| 59 | CR31514 | 125.127 | 0.955 | 0.257 | 3.712 | 2.060E-04 | 6.883E-03 |
| 58 | CG32640 | 1384.636 | 0.950 | 0.164 | 5.792 | 6.959E-09 | 1.608E-06 |
| 57 | CG6094 | 436.089 | 0.880 | 0.200 | 4.402 | 1.071E-05 | 7.175E-04 |
| 56 | CG32537 | 442.757 | 0.870 | 0.204 | 4.276 | 1.899E-05 | 1.120E-03 |
| 54 | CG9960 | 354.645 | 0.867 | 0.166 | 5.229 | 1.705E-07 | 2.331E-05 |
| 55 | Snapin | 354.645 | 0.867 | 0.166 | 5.229 | 1.705E-07 | 2.331E-05 |
| 53 | CG43320 | 156.490 | 0.861 | 0.232 | 3.710 | 2.073E-04 | 6.899E-03 |
| 52 | CG6201 | 280.408 | 0.856 | 0.211 | 4.055 | 5.011E-05 | 2.317E-03 |
| 51 | O-fut2 | 538.309 | 0.855 | 0.234 | 3.654 | 2.586E-04 | 8.189E-03 |
| 50 | Pvf1 | 983.867 | 0.851 | 0.171 | 4.988 | 6.110E-07 | 6.727E-05 |
| 49 | CG9577 | 1129.868 | 0.821 | 0.103 | 7.983 | 1.433E-15 | 2.350E-12 |
| 48 | omd | 1521.315 | 0.816 | 0.217 | 3.760 | 1.700E-04 | 5.935E-03 |
| 47 | CG9723 | 694.005 | 0.808 | 0.188 | 4.291 | 1.779E-05 | 1.069E-03 |
| 46 | sgl | 2121.675 | 0.805 | 0.207 | 3.899 | 9.668E-05 | 3.911E-03 |
| 45 | CG18417 | 283.237 | 0.778 | 0.165 | 4.720 | 2.357E-06 | 2.102E-04 |
| 44 | CG8449 | 958.927 | 0.754 | 0.176 | 4.277 | 1.898E-05 | 1.120E-03 |
| 43 | CG18596 | 761.742 | 0.745 | 0.157 | 4.738 | 2.164E-06 | 1.950E-04 |
| 42 | CG31344 | 326.013 | 0.744 | 0.200 | 3.727 | 1.938E-04 | 6.556E-03 |
| 41 | CG7054 | 933.555 | 0.737 | 0.198 | 3.719 | 1.998E-04 | 6.704E-03 |
| 40 | spict | 771.656 | 0.732 | 0.105 | 6.986 | 2.822E-12 | 2.315E-09 |
| 39 | CG4557 | 995.225 | 0.729 | 0.197 | 3.707 | 2.101E-04 | 6.972E-03 |
| 38 | ash2 | 908.546 | 0.705 | 0.170 | 4.146 | 3.387E-05 | 1.731E-03 |
| 37 | CalpC | 415.048 | 0.703 | 0.162 | 4.336 | 1.449E-05 | 9.286E-04 |
| 36 | CG5590 | 1605.657 | 0.702 | 0.119 | 5.911 | 3.406E-09 | 9.014E-07 |
| 35 | CG7456 | 1107.933 | 0.699 | 0.104 | 6.735 | 1.638E-11 | 1.120E-08 |
| 34 | CG12237 | 1050.910 | 0.692 | 0.175 | 3.963 | 7.387E-05 | 3.108E-03 |
| 33 | sinu | 550.984 | 0.691 | 0.173 | 3.994 | 6.499E-05 | 2.784E-03 |
| 32 | isoQC | 646.011 | 0.685 | 0.134 | 5.109 | 3.243E-07 | 4.125E-05 |
| 31 | Aats-pro | 382.941 | 0.663 | 0.177 | 3.751 | 1.764E-04 | 6.106E-03 |
| 30 | CG8195 | 468.063 | 0.636 | 0.171 | 3.717 | 2.020E-04 | 6.762E-03 |
| 29 | CG18476 | 338.801 | 0.631 | 0.152 | 4.157 | 3.218E-05 | 1.680E-03 |
| 28 | Ef1alpha48D | 412025.142 | 0.623 | 0.157 | 3.969 | 7.208E-05 | 3.048E-03 |
| 27 | trbl | 1262.853 | 0.618 | 0.163 | 3.783 | 1.551E-04 | 5.604E-03 |
| 26 | Trs23 | 222.424 | 0.614 | 0.163 | 3.781 | 1.565E-04 | 5.617E-03 |
| 25 | CG13369 | 1223.838 | 0.600 | 0.160 | 3.758 | 1.710E-04 | 5.944E-03 |
| 24 | Mvl | 2735.297 | 0.582 | 0.150 | 3.877 | 1.056E-04 | 4.187E-03 |
| 23 | Ipk1 | 354.774 | 0.564 | 0.154 | 3.676 | 2.372E-04 | 7.707E-03 |
| 22 | CG12772 | 715.663 | 0.563 | 0.133 | 4.233 | 2.309E-05 | 1.296E-03 |
| 21 | CR44047 | 1042.096 | 0.562 | 0.104 | 5.426 | 5.764E-08 | 9.851E-06 |
| 20 | CG18011 | 385.832 | 0.560 | 0.114 | 4.932 | 8.127E-07 | 8.385E-05 |
| 19 | loj | 1177.930 | 0.550 | 0.118 | 4.682 | 2.845E-06 | 2.470E-04 |
| 18 | pix | 7608.154 | 0.546 | 0.116 | 4.721 | 2.342E-06 | 2.099E-04 |
| 17 | AdSS | 5866.285 | 0.546 | 0.104 | 5.267 | 1.387E-07 | 1.962E-05 |
| 16 | CR43872 | 621.290 | 0.539 | 0.130 | 4.148 | 3.351E-05 | 1.723E-03 |
| 15 | SH3PX1 | 1270.434 | 0.498 | 0.130 | 3.830 | 1.280E-04 | 4.892E-03 |
| 14 | crq | 3459.380 | 0.488 | 0.115 | 4.235 | 2.282E-05 | 1.294E-03 |
| 13 | Ate1 | 1188.668 | 0.482 | 0.108 | 4.471 | 7.777E-06 | 5.500E-04 |
| 12 | P5cr | 465.193 | 0.472 | 0.088 | 5.340 | 9.300E-08 | 1.467E-05 |
| 11 | CG5168 | 2178.283 | 0.446 | 0.117 | 3.798 | 1.457E-04 | 5.312E-03 |
| 10 | qm | 622.400 | 0.443 | 0.104 | 4.242 | 2.216E-05 | 1.271E-03 |
| 9 | Vamp7 | 1555.340 | 0.439 | 0.106 | 4.152 | 3.293E-05 | 1.705E-03 |
| 8 | cert | 1637.451 | 0.437 | 0.108 | 4.054 | 5.043E-05 | 2.324E-03 |
| 7 | AdenoK | 3316.757 | 0.424 | 0.069 | 6.175 | 6.600E-10 | 2.166E-07 |
| 6 | RagC-D | 942.636 | 0.417 | 0.101 | 4.132 | 3.589E-05 | 1.801E-03 |
| 5 | GM130 | 1130.337 | 0.410 | 0.078 | 5.269 | 1.371E-07 | 1.962E-05 |
| 4 | CG8726 | 1579.713 | 0.385 | 0.101 | 3.816 | 1.358E-04 | 5.074E-03 |
| 3 | TfIIA-L | 1828.207 | 0.370 | 0.094 | 3.944 | 8.000E-05 | 3.331E-03 |
| 2 | CR44390 | 494.579 | 0.305 | 0.069 | 4.437 | 9.104E-06 | 6.250E-04 |
| 1 | Gie | 920.899 | 0.288 | 0.075 | 3.847 | 1.196E-04 | 4.617E-03 |
| **INT (compared with NINT)** | | | | | | | |
|  | **Gene symbol** | **baseMean** | **log2FoldChange** | **lfcSE** | **stat** | ***P*value** | ***P*adj** |
| 36 | Cpr65Ax2 | 83.141 | 24.741 | 2.880 | 8.590 | 8.692E-18 | 1.398E-13 |
| 35 | Acp1 | 10432.825 | 8.461 | 1.445 | 5.857 | 4.720E-09 | 1.898E-05 |
| 34 | CR45530 | 2689.245 | 8.202 | 1.330 | 6.169 | 6.860E-10 | 5.518E-06 |
| 33 | CG10591 | 29.419 | 8.126 | 1.630 | 4.985 | 6.195E-07 | 9.060E-04 |
| 32 | Acp65Aa | 1710.694 | 7.277 | 1.438 | 5.060 | 4.185E-07 | 6.732E-04 |
| 31 | CG8736 | 4655.404 | 7.093 | 1.525 | 4.652 | 3.292E-06 | 2.207E-03 |
| 30 | CG7214 | 4545.898 | 7.057 | 1.342 | 5.258 | 1.454E-07 | 2.923E-04 |
| 29 | Cpr92F | 2193.871 | 6.633 | 1.455 | 4.558 | 5.161E-06 | 3.075E-03 |
| 28 | CG13731 | 293.951 | 6.501 | 1.320 | 4.924 | 8.497E-07 | 1.139E-03 |
| 27 | Ccp84Ab | 126.683 | 6.205 | 1.310 | 4.735 | 2.186E-06 | 1.599E-03 |
| 26 | CG13041 | 34.860 | 6.141 | 1.136 | 5.408 | 6.376E-08 | 1.465E-04 |
| 25 | fln | 11224.318 | 6.089 | 1.269 | 4.799 | 1.594E-06 | 1.371E-03 |
| 24 | CR45161 | 3966.659 | 6.022 | 1.258 | 4.786 | 1.705E-06 | 1.371E-03 |
| 23 | Osi7 | 105.886 | 5.907 | 1.326 | 4.455 | 8.402E-06 | 4.661E-03 |
| 22 | CG13060 | 13.996 | 5.603 | 1.299 | 4.313 | 1.612E-05 | 7.264E-03 |
| 21 | CG7203 | 12872.581 | 5.444 | 1.153 | 4.721 | 2.343E-06 | 1.639E-03 |
| 20 | CR45663 | 20.275 | 5.344 | 1.048 | 5.101 | 3.38E-07 | 6.035E-04 |
| 19 | CG15617 | 424.562 | 4.893 | 1.135 | 4.311 | 1.63E-05 | 7.264E-03 |
| 18 | CG13026 | 115.197 | 4.397 | 0.913 | 4.818 | 1.45E-06 | 1.371E-03 |
| 17 | Scp1 | 30418.593 | 3.555 | 0.638 | 5.568 | 2.58E-08 | 6.908E-05 |
| 16 | Osi9 | 33.401 | 2.348 | 0.531 | 4.419 | 9.92E-06 | 5.321E-03 |
| 15 | mag | 3776.051 | 1.836 | 0.383 | 4.790 | 1.67E-06 | 1.371E-03 |
| 14 | CG4461 | 1655.760 | 1.049 | 0.175 | 5.999 | 1.98E-09 | 1.063E-05 |
| 13 | Obp19a | 311.336 | 1.021 | 0.223 | 4.574 | 4.79E-06 | 2.966E-03 |
| 2 | CG7580 | 19980.190 | 0.876 | 0.194 | 4.521 | 6.14E-06 | 3.530E-03 |
| 1 | Mf | 46470.056 | 0.804 | 0.169 | 4.742 | 2.11E-06 | 1.599E-03 |
