# Supplementary material for: Genetic association of intelligence with longevity in Drosophila melanogaster
Source: PLoS One. 2025 Jul 2;20(7):e0325154. doi: 10.1371/journal.pone.0325154 (PMC12221060; doi:10.1371/journal.pone.0325154)
Supplement: S6 Table — (DOCX) [file pone.0325154.s016.docx]

**Supplementary Table 6. The list of the downregulated genes in each population**

| **INT (compared with F_0_)** | | | | | | | |
| --- | --- | --- | --- | --- | --- | --- | --- |
|  | **Gene symbol** | **baseMean** | **log2FoldChange** | **lfcSE** | **stat** | ***P*value** | ***P*adj** |
| 1 | Fbp1 | 380.875 | -7.879 | 1.570 | -5.020 | 5.18E-07 | 0.000223453 |
| 2 | Fbp2 | 226.509 | -5.946 | 1.049 | -5.667 | 1.46E-08 | 9.56E-06 |
| 3 | CG17147 | 350.700 | -4.486 | 0.559 | -8.030 | 9.71E-16 | 2.66E-12 |
| 4 | MtnB | 1153.489 | -3.381 | 0.468 | -7.223 | 5.08E-13 | 6.94E-10 |
| 5 | CG43218 | 123.374 | -2.994 | 0.582 | -5.141 | 2.74E-07 | 0.000124753 |
| 6 | CG15597 | 26.856 | -2.909 | 0.614 | -4.738 | 2.16E-06 | 0.000715026 |
| 7 | CG17145 | 26.105 | -2.345 | 0.524 | -4.471 | 7.79E-06 | 0.002028365 |
| 8 | CG33639 | 157.631 | -1.751 | 0.405 | -4.321 | 1.55E-05 | 0.003643831 |
| 9 | CG12672 | 35.799 | -1.645 | 0.373 | -4.414 | 1.01E-05 | 0.002519725 |
| 10 | CG1304 | 544.845 | -1.552 | 0.201 | -7.723 | 1.14E-14 | 2.66E-11 |
| 11 | CG8628 | 3735.722 | -1.534 | 0.293 | -5.237 | 1.63E-07 | 7.95E-05 |
| 12 | CG11320 | 27.807 | -1.508 | 0.327 | -4.610 | 4.03E-06 | 0.001159209 |
| 13 | CG18107 | 814.713 | -1.448 | 0.241 | -6.007 | 1.89E-09 | 1.47E-06 |
| 14 | CG14854 | 63.351 | -1.358 | 0.316 | -4.294 | 1.75E-05 | 0.003998107 |
| 15 | PGRP-SC2 | 4067.493 | -1.313 | 0.297 | -4.426 | 9.59E-06 | 0.00245859 |
| 16 | CG5999 | 178.952 | -1.230 | 0.280 | -4.396 | 1.10E-05 | 0.002694378 |
| 17 | Ser6 | 892.930 | -1.207 | 0.298 | -4.053 | 5.05E-05 | 0.009631828 |
| 18 | CR43719 | 58.449 | -1.198 | 0.278 | -4.312 | 1.62E-05 | 0.003732435 |
| 19 | udd | 646.532 | -1.105 | 0.235 | -4.709 | 2.49E-06 | 0.000770876 |
| 20 | CG18853 | 1568.274 | -1.000 | 0.176 | -5.673 | 1.40E-08 | 9.56E-06 |
| 21 | mRpL27 | 1313.804 | -0.876 | 0.185 | -4.736 | 2.18E-06 | 0.000715026 |
| 22 | CG3603 | 571.208 | -0.789 | 0.145 | -5.427 | 5.72E-08 | 2.93E-05 |
| 23 | CG6084 | 6772.871 | -0.789 | 0.160 | -4.926 | 8.38E-07 | 0.000327259 |
| 24 | mir-4967 | 86.893 | -0.724 | 0.154 | -4.693 | 2.69E-06 | 0.00081636 |
| 25 | Gip | 1953.270 | -0.714 | 0.161 | -4.419 | 9.93E-06 | 0.002505318 |
| 26 | CG32500 | 1283.226 | -0.701 | 0.138 | -5.065 | 4.08E-07 | 0.000180893 |
| 27 | CG10444 | 1172.959 | -0.699 | 0.163 | -4.280 | 1.87E-05 | 0.004195577 |
| 28 | CG14606 | 194.131 | -0.608 | 0.150 | -4.065 | 4.80E-05 | 0.009288681 |
| 29 | CG33502 | 6281.878 | -0.582 | 0.133 | -4.387 | 1.15E-05 | 0.002770558 |
| 30 | Etf-QO | 2429.156 | -0.409 | 0.089 | -4.577 | 4.72E-06 | 0.001313228 |
| **NINT (compared with F_0_)** | | | | | | | |
|  | **Gene symbol** | **baseMean** | **log2FoldChange** | **lfcSE** | **stat** | ***P*value** | ***P*adj** |
| 1 | Cpr65Ax2 | 83.141 | -24.273 | 2.880 | -8.427 | 3.54E-17 | 1.16E-13 |
| 2 | Acp1 | 10432.825 | -11.322 | 1.445 | -7.837 | 4.61E-15 | 5.82E-12 |
| 3 | Fbp1 | 380.875 | -10.909 | 1.700 | -6.418 | 1.38E-10 | 5.25E-08 |
| 4 | CG5172 | 1602.552 | -10.123 | 1.882 | -5.378 | 7.55E-08 | 1.24E-05 |
| 5 | Acp65Aa | 1710.694 | -10.085 | 1.438 | -7.014 | 2.32E-12 | 2.00E-09 |
| 6 | CG7214 | 4545.898 | -9.852 | 1.342 | -7.341 | 2.12E-13 | 2.48E-10 |
| 7 | CG8736 | 4655.404 | -9.700 | 1.525 | -6.362 | 2.00E-10 | 7.44E-08 |
| 8 | CG17298 | 801.833 | -9.605 | 1.563 | -6.146 | 7.94E-10 | 2.55E-07 |
| 9 | Cpr92F | 2193.871 | -9.593 | 1.455 | -6.593 | 4.32E-11 | 2.26E-08 |
| 10 | CG13065 | 126.642 | -9.563 | 1.749 | -5.466 | 4.59E-08 | 8.19E-06 |
| 11 | CG13043 | 161.138 | -9.458 | 1.800 | -5.253 | 1.49E-07 | 2.09E-05 |
| 12 | CG12998 | 4329.768 | -9.140 | 1.818 | -5.027 | 4.97E-07 | 5.79E-05 |
| 13 | CG11458 | 677.600 | -9.121 | 1.364 | -6.687 | 2.27E-11 | 1.43E-08 |
| 14 | Cpr97Ea | 464.816 | -9.000 | 1.589 | -5.665 | 1.47E-08 | 3.02E-06 |
| 15 | CG31862 | 42.950 | -8.780 | 2.232 | -3.933 | 8.39E-05 | 0.003449276 |
| 16 | CG13731 | 293.951 | -8.748 | 1.320 | -6.630 | 3.36E-11 | 1.97E-08 |
| 17 | fln | 11224.318 | -8.686 | 1.269 | -6.846 | 7.59E-12 | 5.80E-09 |
| 18 | CG13042 | 63.359 | -8.684 | 1.687 | -5.147 | 2.65E-07 | 3.42E-05 |
| 19 | CR45161 | 3966.659 | -8.535 | 1.258 | -6.782 | 1.18E-11 | 8.43E-09 |
| 20 | TwdlN | 26.558 | -8.170 | 2.044 | -3.997 | 6.41E-05 | 0.002760472 |
| 21 | Cpr62Bc | 1823.593 | -8.077 | 1.273 | -6.343 | 2.26E-10 | 7.87E-08 |
| 22 | CG34248 | 15.368 | -8.010 | 1.724 | -4.647 | 3.37E-06 | 0.00027935 |
| 23 | Ccp84Ab | 126.683 | -7.983 | 1.309 | -6.098 | 1.07E-09 | 3.25E-07 |
| 24 | Act88F | 29717.179 | -7.793 | 1.261 | -6.178 | 6.50E-10 | 2.17E-07 |
| 25 | CG7203 | 12872.581 | -7.630 | 1.153 | -6.616 | 3.68E-11 | 2.04E-08 |
| 26 | CG34327 | 1114.960 | -7.542 | 1.404 | -5.371 | 7.83E-08 | 1.26E-05 |
| 27 | Cpr47Ea | 557.072 | -7.415 | 1.128 | -6.573 | 4.92E-11 | 2.41E-08 |
| 28 | CG13041 | 34.860 | -7.387 | 1.132 | -6.524 | 6.85E-11 | 3.04E-08 |
| 29 | Cpr100A | 3112.369 | -7.230 | 1.264 | -5.720 | 1.07E-08 | 2.37E-06 |
| 30 | CG10591 | 29.419 | -6.914 | 1.633 | -4.235 | 2.29E-05 | 0.001294219 |
| 31 | Fbp2 | 226.509 | -6.833 | 1.057 | -6.466 | 1.01E-10 | 4.24E-08 |
| 32 | TpnC4 | 3337.114 | -6.808 | 1.140 | -5.974 | 2.32E-09 | 6.55E-07 |
| 33 | CG15617 | 424.562 | -6.682 | 1.135 | -5.890 | 3.87E-09 | 9.92E-07 |
| 34 | CR45663 | 20.275 | -6.613 | 1.041 | -6.350 | 2.15E-10 | 7.69E-08 |
| 35 | Nplp3 | 16267.039 | -6.564 | 0.992 | -6.614 | 3.73E-11 | 2.04E-08 |
| 36 | Osi15 | 25.512 | -6.529 | 1.797 | -3.634 | 0.000279227 | 0.008683185 |
| 37 | CG13060 | 13.996 | -6.441 | 1.295 | -4.975 | 6.51E-07 | 7.08E-05 |
| 38 | TwdlM | 77.695 | -6.441 | 1.546 | -4.167 | 3.08E-05 | 0.001641136 |
| 39 | Cpr49Ah | 203.432 | -6.318 | 1.277 | -4.949 | 7.48E-07 | 7.86E-05 |
| 40 | Pxd | 249.758 | -6.270 | 1.346 | -4.658 | 3.19E-06 | 0.000267126 |
| 41 | CG2150 | 29.257 | -6.222 | 1.310 | -4.751 | 2.02E-06 | 0.000183095 |
| 42 | CG31904 | 4637.723 | -6.037 | 1.133 | -5.328 | 9.94E-08 | 1.53E-05 |
| 43 | CG14565 | 16.873 | -6.026 | 1.572 | -3.834 | 0.000126282 | 0.004840595 |
| 44 | Cpr49Ae | 4769.455 | -6.014 | 0.985 | -6.108 | 1.01E-09 | 3.13E-07 |
| 45 | TwdlB | 39.348 | -5.964 | 1.412 | -4.224 | 2.40E-05 | 0.00133743 |
| 46 | Ccp84Aa | 44.993 | -5.947 | 1.198 | -4.963 | 6.93E-07 | 7.46E-05 |
| 47 | Or47a | 147.679 | -5.914 | 0.931 | -6.350 | 2.16E-10 | 7.69E-08 |
| 48 | CG16885 | 2322.837 | -5.905 | 1.187 | -4.975 | 6.51E-07 | 7.08E-05 |
| 49 | Osi7 | 105.886 | -5.866 | 1.326 | -4.424 | 9.71E-06 | 0.000660712 |
| 50 | CG11413 | 309.682 | -5.848 | 1.022 | -5.721 | 1.06E-08 | 2.37E-06 |
| 51 | Cpr97Eb | 756.403 | -5.737 | 0.960 | -5.977 | 2.28E-09 | 6.55E-07 |
| 52 | CG34217 | 85.606 | -5.622 | 1.125 | -4.997 | 5.83E-07 | 6.46E-05 |
| 53 | Zasp67 | 592.419 | -5.610 | 1.030 | -5.445 | 5.18E-08 | 9.15E-06 |
| 54 | CG1368 | 242.569 | -5.504 | 0.940 | -5.854 | 4.80E-09 | 1.17E-06 |
| 55 | CG15080 | 288.145 | -5.432 | 1.020 | -5.327 | 9.97E-08 | 1.53E-05 |
| 56 | CG14573 | 2.878 | -5.380 | 1.315 | -4.091 | 4.29E-05 | 0.002050663 |
| 57 | CG4962 | 716.023 | -5.349 | 0.975 | -5.488 | 4.06E-08 | 7.33E-06 |
| 58 | CG16884 | 2158.788 | -5.333 | 1.200 | -4.444 | 8.82E-06 | 0.000610864 |
| 59 | CG34461 | 18.876 | -5.310 | 1.172 | -4.530 | 5.90E-06 | 0.000439614 |
| 60 | St2 | 3.637 | -5.263 | 1.286 | -4.092 | 4.29E-05 | 0.002050663 |
| 61 | Cpr50Cb | 69.627 | -5.234 | 1.026 | -5.101 | 3.39E-07 | 4.24E-05 |
| 62 | kkv | 543.646 | -5.137 | 0.876 | -5.861 | 4.60E-09 | 1.15E-06 |
| 63 | CG2650 | 488.299 | -5.128 | 1.099 | -4.665 | 3.08E-06 | 0.000261898 |
| 64 | CG15212 | 14.328 | -4.925 | 1.228 | -4.009 | 6.10E-05 | 0.002645635 |
| 65 | CG10183 | 4.856 | -4.920 | 1.249 | -3.940 | 8.13E-05 | 0.003360287 |
| 66 | TwdlF | 28.056 | -4.918 | 1.215 | -4.048 | 5.16E-05 | 0.002354162 |
| 67 | CG13643 | 101.581 | -4.874 | 0.881 | -5.532 | 3.16E-08 | 6.05E-06 |
| 68 | CR44492 | 19.817 | -4.854 | 1.160 | -4.183 | 2.88E-05 | 0.001553568 |
| 69 | CR45664 | 16.979 | -4.826 | 0.860 | -5.612 | 2.00E-08 | 4.06E-06 |
| 70 | Lsp1gamma | 176.325 | -4.780 | 1.114 | -4.289 | 1.79E-05 | 0.0010704 |
| 71 | Obp83g | 40.984 | -4.768 | 0.947 | -5.033 | 4.83E-07 | 5.66E-05 |
| 72 | Lcp65Ag3 | 90.204 | -4.681 | 1.163 | -4.026 | 5.68E-05 | 0.002511726 |
| 73 | obst-A | 641.322 | -4.659 | 0.858 | -5.432 | 5.57E-08 | 9.68E-06 |
| 74 | CG13026 | 115.197 | -4.640 | 0.912 | -5.086 | 3.65E-07 | 4.54E-05 |
| 75 | CG13063 | 48.740 | -4.605 | 0.957 | -4.813 | 1.49E-06 | 0.000141809 |
| 76 | CG17147 | 350.700 | -4.582 | 0.557 | -8.221 | 2.02E-16 | 5.53E-13 |
| 77 | l(3)mbn | 41.275 | -4.570 | 1.059 | -4.315 | 1.59E-05 | 0.000989808 |
| 78 | CR44769 | 3.962 | -4.561 | 1.106 | -4.122 | 3.75E-05 | 0.001865199 |
| 79 | CG42792 | 35.315 | -4.551 | 1.089 | -4.180 | 2.91E-05 | 0.001560861 |
| 80 | Lcp65Ag1 | 86.790 | -4.545 | 1.179 | -3.856 | 0.000115451 | 0.004488351 |
| 81 | Cht7 | 344.186 | -4.517 | 0.894 | -5.052 | 4.37E-07 | 5.19E-05 |
| 82 | zye | 420.766 | -4.484 | 0.954 | -4.698 | 2.63E-06 | 0.000231723 |
| 83 | CG13044 | 181.240 | -4.469 | 1.047 | -4.268 | 1.98E-05 | 0.001157994 |
| 84 | prc | 198.315 | -4.456 | 0.849 | -5.247 | 1.55E-07 | 2.15E-05 |
| 85 | Cda4 | 251.446 | -4.365 | 0.899 | -4.855 | 1.21E-06 | 0.000117049 |
| 86 | y | 51.505 | -4.327 | 1.156 | -3.742 | 0.000182915 | 0.00626495 |
| 87 | verm | 2682.579 | -4.302 | 0.843 | -5.104 | 3.32E-07 | 4.19E-05 |
| 88 | Scp1 | 30418.593 | -4.207 | 0.638 | -6.590 | 4.40E-11 | 2.26E-08 |
| 89 | Muc91C | 10.169 | -4.143 | 1.125 | -3.683 | 0.000230214 | 0.007538711 |
| 90 | CG42323 | 49.596 | -4.114 | 0.903 | -4.554 | 5.25E-06 | 0.00039709 |
| 91 | Cpr11A | 31.209 | -4.082 | 0.892 | -4.576 | 4.75E-06 | 0.000365664 |
| 92 | Ccp84Ag | 50.686 | -4.057 | 0.725 | -5.593 | 2.23E-08 | 4.36E-06 |
| 93 | CG13071 | 28.605 | -3.980 | 0.956 | -4.163 | 3.14E-05 | 0.001665111 |
| 94 | Cpr92A | 80.296 | -3.963 | 1.097 | -3.613 | 0.000302406 | 0.009256119 |
| 95 | CG15023 | 9.033 | -3.951 | 0.779 | -5.072 | 3.93E-07 | 4.82E-05 |
| 96 | Lcp65Ag2 | 91.004 | -3.908 | 1.011 | -3.864 | 0.000111628 | 0.004402344 |
| 97 | CR44603 | 19.095 | -3.888 | 0.898 | -4.331 | 1.49E-05 | 0.000938859 |
| 98 | CG16786 | 224.357 | -3.861 | 0.731 | -5.284 | 1.26E-07 | 1.90E-05 |
| 99 | Act79B | 31094.285 | -3.850 | 0.739 | -5.208 | 1.91E-07 | 2.57E-05 |
| 100 | CG43218 | 123.374 | -3.847 | 0.586 | -6.566 | 5.15E-11 | 2.42E-08 |
| 101 | CG13606 | 466.501 | -3.841 | 0.785 | -4.893 | 9.95E-07 | 9.90E-05 |
| 102 | CG15373 | 74.538 | -3.791 | 0.731 | -5.189 | 2.11E-07 | 2.79E-05 |
| 103 | mir-4973 | 6.634 | -3.783 | 0.910 | -4.157 | 3.23E-05 | 0.001679901 |
| 104 | lox2 | 148.910 | -3.768 | 0.583 | -6.458 | 1.06E-10 | 4.25E-08 |
| 105 | CG8483 | 147.560 | -3.686 | 0.856 | -4.305 | 1.67E-05 | 0.001019399 |
| 106 | CG13183 | 57.838 | -3.685 | 0.744 | -4.953 | 7.32E-07 | 7.74E-05 |
| 107 | CG7896 | 134.942 | -3.675 | 0.743 | -4.946 | 7.57E-07 | 7.91E-05 |
| 108 | Gasp | 1688.673 | -3.655 | 0.792 | -4.613 | 3.97E-06 | 0.000317845 |
| 109 | CG34276 | 23.307 | -3.653 | 0.793 | -4.605 | 4.12E-06 | 0.000323638 |
| 110 | CG4000 | 5505.088 | -3.584 | 0.680 | -5.268 | 1.38E-07 | 1.96E-05 |
| 111 | CG5873 | 69.309 | -3.546 | 0.974 | -3.641 | 0.000271589 | 0.008519467 |
| 112 | CG9782 | 359.620 | -3.520 | 0.766 | -4.597 | 4.28E-06 | 0.000334569 |
| 113 | Cht5 | 476.292 | -3.507 | 0.914 | -3.837 | 0.000124732 | 0.004792413 |
| 114 | CG9090 | 10730.172 | -3.445 | 0.638 | -5.401 | 6.62E-08 | 1.11E-05 |
| 115 | serp | 1468.231 | -3.442 | 0.795 | -4.330 | 1.49E-05 | 0.000938859 |
| 116 | CR45024 | 468.621 | -3.440 | 0.685 | -5.025 | 5.04E-07 | 5.83E-05 |
| 117 | CG14752 | 55.303 | -3.426 | 0.760 | -4.506 | 6.60E-06 | 0.000481574 |
| 118 | CG6739 | 74.636 | -3.394 | 0.780 | -4.352 | 1.35E-05 | 0.000873651 |
| 119 | e | 557.440 | -3.296 | 0.803 | -4.106 | 4.02E-05 | 0.001975826 |
| 120 | Cht6 | 562.283 | -3.283 | 0.841 | -3.903 | 9.51E-05 | 0.003870613 |
| 121 | Cpr76Bd | 472.647 | -3.261 | 0.747 | -4.366 | 1.26E-05 | 0.000826826 |
| 122 | CG5391 | 8.794 | -3.254 | 0.865 | -3.762 | 0.00016868 | 0.005925833 |
| 123 | GstS1 | 31880.113 | -3.165 | 0.696 | -4.551 | 5.35E-06 | 0.000402716 |
| 124 | CG34172 | 3630.576 | -3.119 | 0.645 | -4.838 | 1.31E-06 | 0.000126726 |
| 125 | Osi9 | 33.401 | -3.112 | 0.527 | -5.906 | 3.51E-09 | 9.13E-07 |
| 126 | CG15213 | 156.607 | -3.092 | 0.514 | -6.018 | 1.77E-09 | 5.27E-07 |
| 127 | Peritrophin-A | 196.049 | -3.084 | 0.764 | -4.034 | 5.48E-05 | 0.00245716 |
| 128 | TpnC41C | 4218.791 | -3.083 | 0.574 | -5.372 | 7.78E-08 | 1.26E-05 |
| 129 | CG11345 | 12.800 | -3.030 | 0.606 | -4.999 | 5.76E-07 | 6.43E-05 |
| 130 | obst-B | 360.499 | -3.026 | 0.755 | -4.006 | 6.17E-05 | 0.002670033 |
| 131 | CG15597 | 26.856 | -2.998 | 0.608 | -4.934 | 8.07E-07 | 8.38E-05 |
| 132 | CG31775 | 964.355 | -2.954 | 0.817 | -3.616 | 0.000298646 | 0.009192464 |
| 133 | CG4702 | 29.174 | -2.947 | 0.766 | -3.845 | 0.000120535 | 0.004642001 |
| 134 | CG34038 | 5.059 | -2.925 | 0.798 | -3.665 | 0.000247549 | 0.007932192 |
| 135 | Mlc2 | 86950.802 | -2.907 | 0.630 | -4.618 | 3.87E-06 | 0.000313294 |
| 136 | Ctr1B | 1275.470 | -2.903 | 0.572 | -5.075 | 3.87E-07 | 4.77E-05 |
| 137 | Mlc1 | 36333.867 | -2.899 | 0.630 | -4.605 | 4.12E-06 | 0.000323638 |
| 138 | CG42586 | 1219.281 | -2.896 | 0.775 | -3.739 | 0.000185016 | 0.006310551 |
| 139 | CG14566 | 17.394 | -2.841 | 0.730 | -3.891 | 9.98E-05 | 0.00401181 |
| 140 | l(2)34Fc | 2312.409 | -2.837 | 0.700 | -4.052 | 5.08E-05 | 0.002333778 |
| 141 | CG13023 | 19.797 | -2.827 | 0.717 | -3.942 | 8.09E-05 | 0.003359969 |
| 142 | CG13067 | 405.539 | -2.816 | 0.605 | -4.656 | 3.22E-06 | 0.000268551 |
| 143 | CG13305 | 21.272 | -2.807 | 0.524 | -5.354 | 8.62E-08 | 1.37E-05 |
| 144 | TwdlV | 16.062 | -2.802 | 0.746 | -3.756 | 0.000172632 | 0.005987731 |
| 145 | Strn-Mlck | 19126.098 | -2.780 | 0.633 | -4.391 | 1.13E-05 | 0.000752572 |
| 146 | CR44850 | 883.500 | -2.774 | 0.689 | -4.026 | 5.67E-05 | 0.002511726 |
| 147 | CG4835 | 256.202 | -2.759 | 0.560 | -4.929 | 8.25E-07 | 8.46E-05 |
| 148 | Mhc | 163894.787 | -2.751 | 0.638 | -4.315 | 1.60E-05 | 0.000990169 |
| 149 | CG4374 | 52.744 | -2.749 | 0.616 | -4.465 | 8.00E-06 | 0.000560688 |
| 150 | CG17974 | 14.934 | -2.701 | 0.593 | -4.555 | 5.23E-06 | 0.00039709 |
| 151 | CG17777 | 106.109 | -2.696 | 0.561 | -4.805 | 1.55E-06 | 0.000146156 |
| 152 | CG7906 | 6.971 | -2.694 | 0.643 | -4.187 | 2.82E-05 | 0.001531691 |
| 153 | retinin | 1445.162 | -2.689 | 0.647 | -4.158 | 3.22E-05 | 0.001679901 |
| 154 | CG8501 | 60.566 | -2.679 | 0.631 | -4.245 | 2.18E-05 | 0.001256154 |
| 155 | CG15021 | 1251.376 | -2.672 | 0.672 | -3.979 | 6.91E-05 | 0.002936231 |
| 156 | CG18367 | 43.333 | -2.671 | 0.493 | -5.420 | 5.95E-08 | 1.01E-05 |
| 157 | Cpr49Ag | 47.318 | -2.670 | 0.451 | -5.913 | 3.35E-09 | 9.01E-07 |
| 158 | Cpr49Ab | 1921.366 | -2.619 | 0.709 | -3.696 | 0.00021877 | 0.007221603 |
| 159 | CG12009 | 42.288 | -2.598 | 0.704 | -3.690 | 0.000224001 | 0.007364653 |
| 160 | CG13678 | 16.238 | -2.568 | 0.609 | -4.215 | 2.49E-05 | 0.00137546 |
| 161 | CG12672 | 35.799 | -2.564 | 0.383 | -6.692 | 2.20E-11 | 1.43E-08 |
| 162 | CG6118 | 30.264 | -2.541 | 0.699 | -3.634 | 0.000279454 | 0.008683185 |
| 163 | mag | 3776.051 | -2.475 | 0.383 | -6.458 | 1.06E-10 | 4.25E-08 |
| 164 | CG15515 | 152.579 | -2.440 | 0.626 | -3.899 | 9.68E-05 | 0.003911139 |
| 165 | ft | 336.722 | -2.426 | 0.425 | -5.705 | 1.16E-08 | 2.54E-06 |
| 166 | CG33639 | 157.631 | -2.423 | 0.406 | -5.961 | 2.50E-09 | 6.96E-07 |
| 167 | Unc-89 | 10603.089 | -2.405 | 0.561 | -4.284 | 1.84E-05 | 0.001093262 |
| 168 | CR43988 | 14.859 | -2.397 | 0.663 | -3.617 | 0.000298031 | 0.009190773 |
| 169 | CG34375 | 52.581 | -2.383 | 0.636 | -3.749 | 0.000177832 | 0.006129226 |
| 170 | CG7714 | 27.688 | -2.369 | 0.517 | -4.584 | 4.57E-06 | 0.000355105 |
| 171 | CG40198 | 272.290 | -2.360 | 0.605 | -3.903 | 9.51E-05 | 0.003870613 |
| 172 | Npc2d | 689.308 | -2.305 | 0.536 | -4.298 | 1.73E-05 | 0.001049399 |
| 173 | Hsp67Bc | 141.742 | -2.304 | 0.597 | -3.859 | 0.000113685 | 0.004440295 |
| 174 | CG15434 | 243.469 | -2.300 | 0.405 | -5.674 | 1.40E-08 | 2.90E-06 |
| 175 | fj | 88.475 | -2.299 | 0.604 | -3.804 | 0.000142279 | 0.005245468 |
| 176 | ple | 3060.717 | -2.284 | 0.636 | -3.592 | 0.000328 | 0.009801768 |
| 177 | CG34382 | 41.365 | -2.255 | 0.620 | -3.640 | 0.000272306 | 0.008525664 |
| 178 | snmRNA:419 | 37.831 | -2.235 | 0.539 | -4.146 | 3.38E-05 | 0.001730928 |
| 179 | CG12105 | 381.405 | -2.225 | 0.526 | -4.234 | 2.30E-05 | 0.001294219 |
| 180 | Tm2 | 34932.786 | -2.224 | 0.501 | -4.439 | 9.04E-06 | 0.000623418 |
| 181 | Ca-P60A | 59765.827 | -2.211 | 0.336 | -6.571 | 5.00E-11 | 2.41E-08 |
| 182 | CG6472 | 70.559 | -2.195 | 0.416 | -5.278 | 1.31E-07 | 1.92E-05 |
| 183 | Npc2c | 30.333 | -2.185 | 0.552 | -3.957 | 7.59E-05 | 0.003184035 |
| 184 | Cpr62Bb | 353.127 | -2.185 | 0.533 | -4.101 | 4.12E-05 | 0.001994881 |
| 185 | Cda5 | 1433.993 | -2.167 | 0.596 | -3.637 | 0.000275618 | 0.008596561 |
| 186 | CG9297 | 19094.220 | -2.160 | 0.371 | -5.825 | 5.70E-09 | 1.36E-06 |
| 187 | CG14625 | 24.515 | -2.131 | 0.580 | -3.674 | 0.000238892 | 0.00774557 |
| 188 | bt | 31227.463 | -2.113 | 0.495 | -4.268 | 1.97E-05 | 0.001157994 |
| 189 | CR44654 | 8.272 | -2.099 | 0.570 | -3.683 | 0.000230094 | 0.007538711 |
| 190 | Prm | 30453.647 | -2.097 | 0.514 | -4.077 | 4.56E-05 | 0.002151977 |
| 191 | CG4115 | 241.523 | -2.095 | 0.488 | -4.294 | 1.75E-05 | 0.001060781 |
| 192 | CR45591 | 131.166 | -2.089 | 0.554 | -3.768 | 0.000164736 | 0.005837278 |
| 193 | CG14742 | 336.441 | -2.039 | 0.504 | -4.045 | 5.24E-05 | 0.002375237 |
| 194 | kdn | 24995.195 | -2.022 | 0.489 | -4.136 | 3.53E-05 | 0.001783555 |
| 195 | Cpr11B | 48.243 | -2.019 | 0.474 | -4.262 | 2.03E-05 | 0.001183382 |
| 196 | CG32037 | 46.548 | -2.013 | 0.501 | -4.020 | 5.81E-05 | 0.00255042 |
| 197 | CG17211 | 72.824 | -2.004 | 0.465 | -4.313 | 1.61E-05 | 0.000994802 |
| 198 | CG13856 | 64.036 | -1.982 | 0.449 | -4.418 | 9.96E-06 | 0.00067494 |
| 199 | CG32814 | 111.336 | -1.975 | 0.498 | -3.970 | 7.20E-05 | 0.003047654 |
| 200 | CG15615 | 21.278 | -1.971 | 0.443 | -4.452 | 8.50E-06 | 0.000590979 |
| 201 | CG42255 | 169.786 | -1.962 | 0.483 | -4.063 | 4.84E-05 | 0.002257092 |
| 202 | CG14246 | 326.393 | -1.961 | 0.411 | -4.766 | 1.88E-06 | 0.000173162 |
| 203 | ds | 485.608 | -1.929 | 0.493 | -3.916 | 9.02E-05 | 0.00369022 |
| 204 | GstD7 | 38.976 | -1.897 | 0.399 | -4.756 | 1.98E-06 | 0.000180102 |
| 205 | CG45218 | 2061.974 | -1.879 | 0.487 | -3.859 | 0.000113708 | 0.004440295 |
| 206 | Abl | 9487.098 | -1.872 | 0.432 | -4.331 | 1.48E-05 | 0.000938859 |
| 207 | CG8927 | 318.518 | -1.866 | 0.519 | -3.596 | 0.00032324 | 0.009699442 |
| 208 | CG9192 | 63.313 | -1.866 | 0.398 | -4.687 | 2.78E-06 | 0.000242305 |
| 209 | CG44142 | 856.529 | -1.859 | 0.406 | -4.579 | 4.66E-06 | 0.000360793 |
| 210 | Gpo-1 | 6669.610 | -1.858 | 0.508 | -3.655 | 0.000256709 | 0.008146162 |
| 211 | CG9642 | 13.492 | -1.842 | 0.477 | -3.859 | 0.000113944 | 0.004440295 |
| 212 | Sh | 2284.912 | -1.840 | 0.371 | -4.958 | 7.14E-07 | 7.60E-05 |
| 213 | mthl13 | 39.366 | -1.836 | 0.480 | -3.824 | 0.00013144 | 0.00499166 |
| 214 | dao | 230.481 | -1.831 | 0.346 | -5.287 | 1.24E-07 | 1.89E-05 |
| 215 | CG9095 | 160.166 | -1.820 | 0.479 | -3.803 | 0.000143136 | 0.005265232 |
| 216 | MtnB | 1153.489 | -1.814 | 0.467 | -3.886 | 0.000101746 | 0.004061418 |
| 217 | CG12483 | 82.782 | -1.813 | 0.437 | -4.150 | 3.33E-05 | 0.001717579 |
| 218 | mir-4982 | 13.071 | -1.795 | 0.426 | -4.214 | 2.51E-05 | 0.00137546 |
| 219 | Act87E | 22773.479 | -1.791 | 0.396 | -4.517 | 6.26E-06 | 0.000462809 |
| 220 | Tm1 | 46829.064 | -1.778 | 0.475 | -3.744 | 0.000181059 | 0.006214345 |
| 221 | RunxB | 18.357 | -1.769 | 0.455 | -3.888 | 0.000101204 | 0.004049659 |
| 222 | CG11162 | 87.267 | -1.760 | 0.361 | -4.876 | 1.08E-06 | 0.000106901 |
| 223 | RyR | 7231.416 | -1.745 | 0.453 | -3.854 | 0.000116341 | 0.004512283 |
| 224 | CG2022 | 269.237 | -1.740 | 0.448 | -3.888 | 0.000100964 | 0.004049659 |
| 225 | Obp28a | 238.483 | -1.722 | 0.323 | -5.334 | 9.62E-08 | 1.50E-05 |
| 226 | CG34155 | 240.827 | -1.714 | 0.475 | -3.607 | 0.000309728 | 0.009409998 |
| 227 | CG11321 | 2825.057 | -1.708 | 0.424 | -4.026 | 5.68E-05 | 0.002511726 |
| 228 | Cpr47Ee | 134.351 | -1.690 | 0.399 | -4.238 | 2.26E-05 | 0.001289472 |
| 229 | pncr004:X | 53.827 | -1.680 | 0.412 | -4.073 | 4.64E-05 | 0.002180688 |
| 230 | CG14854 | 63.351 | -1.667 | 0.316 | -5.281 | 1.29E-07 | 1.92E-05 |
| 231 | CG1537 | 26.329 | -1.664 | 0.407 | -4.090 | 4.31E-05 | 0.002050663 |
| 232 | tipE | 298.186 | -1.662 | 0.382 | -4.346 | 1.38E-05 | 0.000893755 |
| 233 | b | 732.964 | -1.659 | 0.406 | -4.089 | 4.34E-05 | 0.002057179 |
| 234 | CG7631 | 221.213 | -1.657 | 0.327 | -5.066 | 4.06E-07 | 4.93E-05 |
| 235 | CG6329 | 974.041 | -1.647 | 0.305 | -5.395 | 6.85E-08 | 1.14E-05 |
| 236 | CG1304 | 544.845 | -1.641 | 0.200 | -8.187 | 2.67E-16 | 6.17E-13 |
| 237 | Cpr65Au | 1781.923 | -1.639 | 0.348 | -4.712 | 2.46E-06 | 0.000218021 |
| 238 | CR44997 | 19.182 | -1.631 | 0.437 | -3.731 | 0.000190877 | 0.006483488 |
| 239 | CG9682 | 2223.606 | -1.613 | 0.381 | -4.232 | 2.31E-05 | 0.001295621 |
| 240 | CG1732 | 608.954 | -1.612 | 0.427 | -3.772 | 0.000162143 | 0.00577033 |
| 241 | Spn31A | 156.946 | -1.612 | 0.312 | -5.162 | 2.44E-07 | 3.17E-05 |
| 242 | CR45226 | 30.911 | -1.604 | 0.326 | -4.917 | 8.81E-07 | 8.98E-05 |
| 243 | snoRNA:Me18S-A1061 | 22.478 | -1.579 | 0.360 | -4.385 | 1.16E-05 | 0.000770709 |
| 244 | CG14298 | 96.566 | -1.567 | 0.387 | -4.049 | 5.14E-05 | 0.002353294 |
| 245 | Cyt-c-p | 26204.624 | -1.562 | 0.380 | -4.116 | 3.86E-05 | 0.001905433 |
| 246 | salm | 425.846 | -1.541 | 0.359 | -4.291 | 1.78E-05 | 0.001069387 |
| 247 | CG9468 | 6770.701 | -1.541 | 0.406 | -3.799 | 0.000145439 | 0.005311543 |
| 248 | CG3713 | 150.634 | -1.540 | 0.420 | -3.666 | 0.000246586 | 0.007932192 |
| 249 | CG18136 | 126.055 | -1.510 | 0.375 | -4.024 | 5.72E-05 | 0.002517687 |
| 250 | Ugt86Dg | 48.350 | -1.507 | 0.368 | -4.094 | 4.24E-05 | 0.002042182 |
| 251 | CG17374 | 6012.289 | -1.504 | 0.364 | -4.131 | 3.61E-05 | 0.001805756 |
| 252 | Actn | 13137.543 | -1.493 | 0.408 | -3.663 | 0.000249658 | 0.007965439 |
| 253 | snoRNA:Or-CD4 | 24.263 | -1.491 | 0.363 | -4.104 | 4.06E-05 | 0.001977334 |
| 254 | CG10581 | 135.951 | -1.484 | 0.403 | -3.682 | 0.000231589 | 0.007568627 |
| 255 | CG32407 | 787.724 | -1.483 | 0.331 | -4.477 | 7.58E-06 | 0.000540577 |
| 256 | tmod | 6029.938 | -1.480 | 0.356 | -4.162 | 3.15E-05 | 0.001666046 |
| 257 | para | 2587.828 | -1.472 | 0.378 | -3.896 | 9.77E-05 | 0.003937607 |
| 258 | CG8628 | 3735.722 | -1.470 | 0.293 | -5.020 | 5.18E-07 | 5.94E-05 |
| 259 | CG42319 | 1662.157 | -1.469 | 0.300 | -4.895 | 9.84E-07 | 9.84E-05 |
| 260 | Gpdh | 18741.562 | -1.465 | 0.363 | -4.031 | 5.55E-05 | 0.002480623 |
| 261 | Mical | 10082.424 | -1.458 | 0.346 | -4.215 | 2.50E-05 | 0.00137546 |
| 262 | haf | 470.574 | -1.452 | 0.397 | -3.660 | 0.000252488 | 0.008027744 |
| 263 | Hsc70-1 | 2713.106 | -1.451 | 0.402 | -3.608 | 0.000309147 | 0.009409783 |
| 264 | Dop2R | 834.297 | -1.430 | 0.386 | -3.706 | 0.000210367 | 0.006972279 |
| 265 | CG14459 | 57.593 | -1.417 | 0.371 | -3.821 | 0.000132859 | 0.005007647 |
| 266 | CG4461 | 1655.760 | -1.405 | 0.175 | -8.037 | 9.22E-16 | 1.68E-12 |
| 267 | CG9486 | 73.162 | -1.391 | 0.321 | -4.333 | 1.47E-05 | 0.000938859 |
| 268 | TM4SF | 786.462 | -1.342 | 0.311 | -4.317 | 1.58E-05 | 0.000986763 |
| 269 | CR44986 | 2139.431 | -1.342 | 0.373 | -3.600 | 0.000317929 | 0.009605776 |
| 270 | nrm | 1370.728 | -1.328 | 0.349 | -3.801 | 0.000143896 | 0.005280574 |
| 271 | pHCl | 1205.234 | -1.327 | 0.369 | -3.596 | 0.000323394 | 0.009699442 |
| 272 | CG42337 | 268.494 | -1.320 | 0.356 | -3.711 | 0.000206487 | 0.006885426 |
| 273 | CG8665 | 700.113 | -1.318 | 0.358 | -3.680 | 0.000233381 | 0.007612016 |
| 274 | CG6439 | 9964.128 | -1.318 | 0.347 | -3.792 | 0.000149443 | 0.005436264 |
| 275 | CG1136 | 616.144 | -1.306 | 0.347 | -3.761 | 0.000169063 | 0.005926586 |
| 276 | CG18536 | 125.904 | -1.299 | 0.290 | -4.482 | 7.39E-06 | 0.000529138 |
| 277 | Frq2 | 679.405 | -1.294 | 0.290 | -4.460 | 8.21E-06 | 0.000573173 |
| 278 | CG5999 | 178.952 | -1.291 | 0.279 | -4.633 | 3.61E-06 | 0.000295125 |
| 279 | luna | 835.944 | -1.284 | 0.319 | -4.028 | 5.62E-05 | 0.002506513 |
| 280 | danr | 55.690 | -1.273 | 0.307 | -4.142 | 3.45E-05 | 0.001745681 |
| 281 | MsR2 | 122.995 | -1.269 | 0.321 | -3.951 | 7.79E-05 | 0.003259936 |
| 282 | CG14482 | 8278.558 | -1.239 | 0.303 | -4.087 | 4.38E-05 | 0.00206998 |
| 283 | nrv3 | 4476.249 | -1.236 | 0.310 | -3.990 | 6.60E-05 | 0.002810344 |
| 284 | Zasp52 | 12960.715 | -1.225 | 0.303 | -4.039 | 5.37E-05 | 0.002412261 |
| 285 | CG3841 | 1025.673 | -1.218 | 0.297 | -4.105 | 4.04E-05 | 0.001977334 |
| 286 | Fhos | 4016.235 | -1.201 | 0.259 | -4.644 | 3.42E-06 | 0.000281596 |
| 287 | CG13003 | 478.513 | -1.191 | 0.315 | -3.777 | 0.000158707 | 0.00566031 |
| 288 | CG7580 | 19980.190 | -1.189 | 0.194 | -6.136 | 8.48E-10 | 2.68E-07 |
| 289 | CR45712 | 199.518 | -1.186 | 0.315 | -3.763 | 0.000167724 | 0.005904887 |
| 290 | tutl | 1215.467 | -1.180 | 0.317 | -3.726 | 0.000194564 | 0.006567934 |
| 291 | CG40472 | 4837.485 | -1.174 | 0.293 | -4.013 | 6.00E-05 | 0.002611899 |
| 292 | antdh | 272.432 | -1.158 | 0.308 | -3.760 | 0.000169773 | 0.005935025 |
| 293 | CG43149 | 61.030 | -1.140 | 0.268 | -4.255 | 2.09E-05 | 0.001205944 |
| 294 | CG11876 | 11223.836 | -1.140 | 0.237 | -4.811 | 1.50E-06 | 0.000142095 |
| 295 | CG33510 | 33.672 | -1.130 | 0.309 | -3.653 | 0.00025946 | 0.008201742 |
| 296 | CG1674 | 4132.038 | -1.125 | 0.285 | -3.945 | 7.97E-05 | 0.003325293 |
| 297 | CG42346 | 315.392 | -1.122 | 0.297 | -3.782 | 0.00015583 | 0.005608867 |
| 298 | CG3560 | 10390.486 | -1.121 | 0.296 | -3.786 | 0.000153155 | 0.005546724 |
| 299 | CG7781 | 933.660 | -1.119 | 0.294 | -3.806 | 0.000141172 | 0.005245468 |
| 300 | udd | 646.532 | -1.117 | 0.234 | -4.767 | 1.87E-06 | 0.000173162 |
| 301 | CG33296 | 109.552 | -1.111 | 0.306 | -3.633 | 0.000280078 | 0.008686137 |
| 302 | ATPsyn-Cf6 | 22331.333 | -1.096 | 0.301 | -3.642 | 0.000270346 | 0.00849673 |
| 303 | CG4692 | 12668.329 | -1.091 | 0.260 | -4.198 | 2.70E-05 | 0.001475859 |
| 304 | CG6463 | 1981.578 | -1.086 | 0.267 | -4.068 | 4.73E-05 | 0.002218181 |
| 305 | CR43957 | 52.352 | -1.086 | 0.262 | -4.142 | 3.44E-05 | 0.001745681 |
| 306 | CG18107 | 814.713 | -1.085 | 0.240 | -4.515 | 6.32E-06 | 0.000465068 |
| 307 | mir-929 | 305.943 | -1.077 | 0.299 | -3.601 | 0.000317479 | 0.009605776 |
| 308 | CoVb | 9675.183 | -1.075 | 0.269 | -4.002 | 6.27E-05 | 0.002706187 |
| 309 | CG32230 | 12291.814 | -1.074 | 0.204 | -5.273 | 1.34E-07 | 1.95E-05 |
| 310 | Neb-cGP | 10111.835 | -1.060 | 0.258 | -4.109 | 3.98E-05 | 0.001961529 |
| 311 | CG12119 | 671.301 | -1.058 | 0.254 | -4.173 | 3.01E-05 | 0.001607399 |
| 312 | CG9034 | 2585.079 | -1.056 | 0.285 | -3.704 | 0.000212068 | 0.007014476 |
| 313 | CG40002 | 6445.759 | -1.050 | 0.261 | -4.018 | 5.87E-05 | 0.002569897 |
| 314 | bi | 269.895 | -1.047 | 0.281 | -3.724 | 0.00019635 | 0.006601049 |
| 315 | CG3192 | 3975.664 | -1.042 | 0.275 | -3.791 | 0.000149948 | 0.005442564 |
| 316 | CG5548 | 3355.738 | -1.042 | 0.230 | -4.527 | 5.98E-06 | 0.00044396 |
| 317 | CG33521 | 3541.515 | -1.039 | 0.260 | -3.990 | 6.59E-05 | 0.002810344 |
| 318 | Rh50 | 677.636 | -1.039 | 0.252 | -4.123 | 3.74E-05 | 0.001864918 |
| 319 | CoVIII | 7461.435 | -1.037 | 0.271 | -3.821 | 0.000133081 | 0.005007647 |
| 320 | CG7630 | 13105.576 | -1.031 | 0.234 | -4.405 | 1.06E-05 | 0.000710009 |
| 321 | mRpL27 | 1313.804 | -1.022 | 0.185 | -5.532 | 3.17E-08 | 6.05E-06 |
| 322 | Pfk | 6416.630 | -1.018 | 0.267 | -3.818 | 0.00013434 | 0.005031906 |
| 323 | CG10320 | 10086.579 | -1.012 | 0.233 | -4.345 | 1.39E-05 | 0.000897111 |
| 324 | CG6733 | 1194.402 | -1.010 | 0.216 | -4.664 | 3.10E-06 | 0.000262432 |
| 325 | snoRNA:Me28S-G2703c | 34.428 | -0.997 | 0.261 | -3.822 | 0.000132243 | 0.005007647 |
| 326 | CG12859 | 1997.149 | -0.992 | 0.244 | -4.064 | 4.83E-05 | 0.00225581 |
| 327 | CG14661 | 1926.116 | -0.982 | 0.239 | -4.103 | 4.07E-05 | 0.001977334 |
| 328 | CG9306 | 5319.977 | -0.982 | 0.258 | -3.807 | 0.00014042 | 0.00523575 |
| 329 | CheB38c | 76.363 | -0.980 | 0.269 | -3.642 | 0.000270105 | 0.00849673 |
| 330 | CG34439 | 3178.814 | -0.976 | 0.214 | -4.571 | 4.86E-06 | 0.000372874 |
| 331 | CG3321 | 10906.747 | -0.966 | 0.236 | -4.090 | 4.31E-05 | 0.002050663 |
| 332 | CheA7a | 273.572 | -0.949 | 0.248 | -3.830 | 0.000128223 | 0.00489215 |
| 333 | levy | 11674.570 | -0.937 | 0.251 | -3.730 | 0.000191525 | 0.00649207 |
| 334 | cype | 9871.802 | -0.928 | 0.253 | -3.665 | 0.000247527 | 0.007932192 |
| 335 | bnb | 3647.168 | -0.926 | 0.247 | -3.746 | 0.000179404 | 0.006170455 |
| 336 | CG15629 | 120.410 | -0.918 | 0.214 | -4.289 | 1.79E-05 | 0.0010704 |
| 337 | CoVIIc | 14295.508 | -0.912 | 0.228 | -3.994 | 6.49E-05 | 0.002783727 |
| 338 | CG18853 | 1568.274 | -0.904 | 0.176 | -5.135 | 2.82E-07 | 3.61E-05 |
| 339 | CoVa | 13253.721 | -0.886 | 0.202 | -4.380 | 1.18E-05 | 0.000783706 |
| 340 | CG3214 | 4054.794 | -0.885 | 0.216 | -4.099 | 4.15E-05 | 0.002003868 |
| 341 | CG31323 | 128.041 | -0.880 | 0.233 | -3.780 | 0.00015695 | 0.00562211 |
| 342 | CoIV | 15422.205 | -0.877 | 0.227 | -3.863 | 0.000111979 | 0.004405572 |
| 343 | CG6432 | 1232.400 | -0.877 | 0.175 | -5.016 | 5.28E-07 | 6.01E-05 |
| 344 | snmRNA:359 | 265.589 | -0.861 | 0.238 | -3.613 | 0.000302168 | 0.009256119 |
| 345 | l(2)06225 | 20016.603 | -0.860 | 0.212 | -4.055 | 5.01E-05 | 0.00231731 |
| 346 | CG14606 | 194.131 | -0.854 | 0.149 | -5.729 | 1.01E-08 | 2.30E-06 |
| 347 | CG7712 | 3230.884 | -0.852 | 0.228 | -3.736 | 0.000187277 | 0.006374421 |
| 348 | CG5177 | 2622.150 | -0.845 | 0.225 | -3.750 | 0.000177031 | 0.006114467 |
| 349 | CG15822 | 454.130 | -0.842 | 0.224 | -3.766 | 0.000166173 | 0.005862883 |
| 350 | CG8012 | 1199.215 | -0.842 | 0.219 | -3.852 | 0.000117247 | 0.00453667 |
| 351 | Zasp66 | 14150.688 | -0.841 | 0.220 | -3.819 | 0.000134098 | 0.005031906 |
| 352 | Ant2 | 4506.151 | -0.837 | 0.216 | -3.881 | 0.000104038 | 0.004142816 |
| 353 | CG10219 | 4011.205 | -0.835 | 0.173 | -4.821 | 1.43E-06 | 0.00013723 |
| 354 | CG31446 | 367.644 | -0.827 | 0.225 | -3.669 | 0.000243266 | 0.007856349 |
| 355 | CG44242 | 1315.064 | -0.827 | 0.199 | -4.159 | 3.20E-05 | 0.001679901 |
| 356 | NP15.6 | 3000.800 | -0.823 | 0.227 | -3.620 | 0.000294781 | 0.009107675 |
| 357 | CG1970 | 6029.785 | -0.822 | 0.220 | -3.740 | 0.00018371 | 0.006279069 |
| 358 | Mlp84B | 10468.050 | -0.821 | 0.186 | -4.407 | 1.05E-05 | 0.000707676 |
| 359 | Prx5 | 5412.786 | -0.819 | 0.141 | -5.814 | 6.09E-09 | 1.43E-06 |
| 360 | l(1)G0136 | 1129.327 | -0.806 | 0.179 | -4.493 | 7.02E-06 | 0.000505705 |
| 361 | Prosalpha1 | 9288.376 | -0.805 | 0.187 | -4.306 | 1.66E-05 | 0.001016007 |
| 362 | CG3621 | 2967.121 | -0.802 | 0.188 | -4.260 | 2.04E-05 | 0.001188056 |
| 363 | CG11617 | 195.320 | -0.800 | 0.190 | -4.216 | 2.49E-05 | 0.00137546 |
| 364 | CG30382 | 9480.904 | -0.797 | 0.193 | -4.135 | 3.55E-05 | 0.001788484 |
| 365 | CG17026 | 259.411 | -0.787 | 0.182 | -4.327 | 1.51E-05 | 0.000950196 |
| 366 | mir-4967 | 86.893 | -0.786 | 0.151 | -5.203 | 1.96E-07 | 2.61E-05 |
| 367 | Mf | 46470.056 | -0.781 | 0.169 | -4.605 | 4.12E-06 | 0.000323638 |
| 368 | CoVIb | 17934.595 | -0.773 | 0.211 | -3.670 | 0.000242142 | 0.007835468 |
| 369 | l(2)03659 | 93.263 | -0.769 | 0.162 | -4.756 | 1.97E-06 | 0.000180102 |
| 370 | Fili | 126.803 | -0.764 | 0.208 | -3.666 | 0.00024682 | 0.007932192 |
| 371 | CG5023 | 2279.186 | -0.756 | 0.209 | -3.611 | 0.000304835 | 0.009313067 |
| 372 | Argk | 83791.819 | -0.724 | 0.192 | -3.767 | 0.000165406 | 0.005848375 |
| 373 | Cralbp | 162.549 | -0.712 | 0.176 | -4.043 | 5.28E-05 | 0.002381049 |
| 374 | kcc | 4123.316 | -0.703 | 0.195 | -3.610 | 0.000305823 | 0.009325891 |
| 375 | HmgZ | 4549.367 | -0.693 | 0.126 | -5.516 | 3.46E-08 | 6.45E-06 |
| 376 | CG10550 | 1486.181 | -0.677 | 0.162 | -4.181 | 2.91E-05 | 0.001560861 |
| 377 | CG17109 | 646.295 | -0.674 | 0.168 | -4.017 | 5.89E-05 | 0.002571726 |
| 378 | CG17027 | 777.825 | -0.667 | 0.185 | -3.600 | 0.000318674 | 0.009610585 |
| 379 | CG7231 | 969.325 | -0.652 | 0.180 | -3.614 | 0.000301538 | 0.009256119 |
| 380 | CG30054 | 397.069 | -0.638 | 0.177 | -3.604 | 0.000313701 | 0.00951308 |
| 381 | CG9249 | 613.615 | -0.630 | 0.145 | -4.357 | 1.32E-05 | 0.000859497 |
| 382 | CG4848 | 1272.899 | -0.610 | 0.160 | -3.821 | 0.000132678 | 0.005007647 |
| 383 | CG17029 | 1646.513 | -0.599 | 0.158 | -3.781 | 0.000155897 | 0.005608867 |
| 384 | CG14431 | 181.687 | -0.585 | 0.157 | -3.724 | 0.000195859 | 0.006598087 |
| 385 | CG7215 | 2466.968 | -0.582 | 0.137 | -4.236 | 2.28E-05 | 0.001294219 |
| 386 | CG32500 | 1283.226 | -0.520 | 0.138 | -3.769 | 0.000163859 | 0.005818771 |
| 387 | slmo | 7194.270 | -0.516 | 0.140 | -3.679 | 0.000234489 | 0.00763299 |
| 388 | CG33502 | 6281.878 | -0.482 | 0.133 | -3.632 | 0.000281211 | 0.008704796 |
| 389 | CG34179 | 3718.006 | -0.478 | 0.126 | -3.805 | 0.000141626 | 0.005245468 |
| 390 | CG31812 | 418.567 | -0.463 | 0.122 | -3.777 | 0.000158467 | 0.00566031 |
| 391 | AMPdeam | 5306.512 | -0.426 | 0.095 | -4.474 | 7.67E-06 | 0.000544836 |
| 392 | P5CDh1 | 5050.834 | -0.415 | 0.103 | -4.047 | 5.19E-05 | 0.002360572 |
| 393 | CaMKII | 4796.098 | -0.407 | 0.107 | -3.801 | 0.000144197 | 0.005280574 |
| 394 | CG9603 | 10340.614 | -0.384 | 0.090 | -4.257 | 2.07E-05 | 0.00119971 |
| 395 | CG8993 | 1557.517 | -0.358 | 0.089 | -4.043 | 5.28E-05 | 0.002381049 |
| 396 | tacc | 6664.034 | -0.300 | 0.049 | -6.184 | 6.26E-10 | 2.14E-07 |
| 397 | PRL-1 | 4967.644 | -0.268 | 0.064 | -4.214 | 2.51E-05 | 0.00137546 |
| **INT (compared with NINT)** | | | | | | | |
|  | **Gene symbol** | **baseMean** | **log2FoldChange** | **lfcSE** | **stat** | ***P*value** | ***P*adj** |
| 1 | sha | 1929.002 | -7.693 | 1.327 | -5.798 | 6.72E-09 | 2.16E-05 |
| 2 | Or47b | 301.666 | -7.327 | 1.513 | -4.842 | 1.29E-06 | 0.001371225 |
| 3 | Obp47b | 149.550 | -6.828 | 1.577 | -4.330 | 1.49E-05 | 0.007060236 |
| 4 | nompA | 1136.894 | -4.635 | 1.013 | -4.575 | 4.75E-06 | 0.002966214 |
| 5 | CG7741 | 2210.587 | -3.318 | 0.761 | -4.357 | 1.32E-05 | 0.006617489 |
| 6 | CR44192 | 618.494 | -1.669 | 0.341 | -4.894 | 9.90E-07 | 0.001224877 |
| 7 | CG9547 | 716.607 | -0.570 | 0.119 | -4.793 | 1.64E-06 | 0.001371225 |
| 8 | spict | 771.656 | -0.507 | 0.104 | -4.852 | 1.22E-06 | 0.001371225 |
| 9 | yip2 | 3603.651 | -0.405 | 0.093 | -4.343 | 1.40E-05 | 0.006848324 |
| 10 | Nsf2 | 2055.828 | -0.344 | 0.078 | -4.380 | 1.19E-05 | 0.006172458 |
