# Supplementary material for: Genetic association of intelligence with longevity in Drosophila melanogaster
Source: PLoS One. 2025 Jul 2;20(7):e0325154. doi: 10.1371/journal.pone.0325154 (PMC12221060; doi:10.1371/journal.pone.0325154)
Supplement: S7 Table — (DOCX) [file pone.0325154.s017.docx]

**Supplementary Table 7. The list of the Kyoto Encyclopedia of Genes and Genomes (KEGG) pathway**

**INT (compared with F_0_)**

| **ID** | **Description** | **setSize** | **enrichmentScore** | **NES** | **pvalue** | **p.adjust** | **qvalues** | **rank** | **leading_edge** | **core_enrichment** |
| --- | --- | --- | --- | --- | --- | --- | --- | --- | --- | --- |
| dme01200 | Carbon  metabolism | 93 | -0.560521896 | -1.877177655 | 0.0001 | 0.006513694 | 0.005713766 | 3658 | tags=65%, list=30%, signal=45% | 42626/34414/39986/34474/35886/34554/44291/42832/32292/39899/326220/41869/32974/41360/246599/32013/33351/39085/45875/3772542/33475/41269/31185/39988/3772692/43102/42620/42185/40409/47173/40445/33459/41324/43191/317974/117364/35728/46246/37900/34198/38358/39469/43582/33461/31407/41787/39945/317829/43447/39470/40012/36839/36060/42621/41253/37541/41468/36904/3771779/31579 |
| dme03008 | Ribosome  biogenesis  in eukaryotes | 74 | -0.546414784 | -1.767741361 | 0.0001 | 0.006513694 | 0.005713766 | 196 | tags=18%, list=2%, signal=18% | 2768873/2768872/2768902/2768897/2768904/2768873/2768905/7354447/2768899/2768898/117463/2768899/2768873/2768873 |
| dme01230 | Biosynthesis of amino  acids | 52 | -0.618350843 | -1.883826793 | 0.0001 | 0.006513694 | 0.005713766 | 3398 | tags=69%, list=28%, signal=50% | 34554/44291/42732/32292/42729/39878/246599/41274/33351/39085/33475/38871/41269/43102/42620/40409/33459/42730/35728/46246/37900/42728/43582/33461/41787/39945/317829/43447/40012/42783/36060/42731/42621/41468/36904/31579 |
| dme04137 | Mitophagy - animal | 46 | -0.613791737 | -1.832630836 | 0.0004 | 0.014740917 | 0.012930629 | 196 | tags=30%, list=2%, signal=30% | 2768873/2768872/33797/2768902/2768897/2768904/2768873/2768905/7354447/2768899/2768898/117463/2768899/2768873/2768873 |
| dme03010 | Ribosome | 132 | 0.448846615 | 1.743771078 | 0.0008 | 0.020886076 | 0.018321119 | 3314 | tags=58%, list=27%, signal=43% | 50381/36570/38397/31009/42464/31483/41200/36851/45329/31700/43103/37628/31106/35988/34412/3355144/43169/32635/40451/43723/31897/38794/34625/44783/39088/36321/35453/31613/34329/32953/36985/43573/39480/44150/34526/39484/33629/43594/42470/49425/34352/34420/40091/3354918/34098/36855/43349/42061/40654/33487/40060/37430/44059/37589/33654/35098/41120/31228/42761/41372/40687/43532/31588/41807/38983/32700/40980/37292/33214/36576/251466/44326/34149/41347/37235/38208/3355124 |
| dme00010 | Glycolysis / Gluconeogenesis | 42 | -0.603670663 | -1.778578832 | 0.0010 | 0.023026316 | 0.020198523 | 2642 | tags=57%, list=22%, signal=45% | 33351/45875/33475/39988/42620/34779/40409/33459/32345/43191/117364/35728/46246/43582/36510/261603/33461/31407/41787/39945/43447/36060/42621/36904 |
| dme00620 | Pyruvate  metabolism | 34 | -0.636442254 | -1.800662366 | 0.0014 | 0.025808133 | 0.022638714 | 2762 | tags=59%, list=23%, signal=46% | 49997/3772542/33475/39988/3772692/42620/42185/40409/47173/5740441/36556/39469/36510/31407/41787/39945/39470/36839/42621/31830 |
| dme04310 | Wnt  signaling pathway | 93 | -0.492154573 | -1.648216732 | 0.0018 | 0.029920491 | 0.026246045 | 1533 | tags=23%, list=13%, signal=20% | 37300/37909/35065/12798588/37763/32188/34819/2768873/42574/2768872/2768902/2768897/2768904/2768873/2768905/7354447/2768899/2768898/117463/2768899/2768873/2768873 |
| dme04140 | Autophagy - animal | 93 | 0.456765579 | 1.68189576 | 0.0032 | 0.046593717 | 0.040871681 | 2251 | tags=26%, list=19%, signal=21% | 326175/326174/34493/326263/34494/42094/33330/3354888/42692/31666/42096/45268/39150/40201/42095/318725/43991/37733/41957/35793/33283/42358/40653/40278 |
| dme00051 | Fructose and mannose  metabolism | 24 | -0.6352817 | -1.674137086 | 0.0075 | 0.098584098 | 0.086477279 | 2700 | tags=62%, list=22%, signal=49% | 35586/38463/40204/45875/39280/40203/38462/39305/40836/43191/117364/43582/19988923/39279/36060 |
| dme01200 | Carbon  metabolism | 93 | -0.560521896 | -1.877177655 | 0.0001 | 0.006513694 | 0.005713766 | 3658 | tags=65%, list=30%, signal=45% | 42626/34414/39986/34474/35886/34554/44291/42832/32292/39899/326220/41869/32974/41360/246599/32013/33351/39085/45875/3772542/33475/41269/31185/39988/3772692/43102/42620/42185/40409/47173/40445/33459/41324/43191/317974/117364/35728/46246/37900/34198/38358/39469/43582/33461/31407/41787/39945/317829/43447/39470/40012/36839/36060/42621/41253/37541/41468/36904/3771779/31579 |

**NINT (compared with F_0_)**

| **ID** | **Description** | **setSize** | **enrichmentScore** | **NES** | **pvalue** | **p.adjust** | **qvalues** | **rank** | **leading_edge** | **core_enrichment** |
| --- | --- | --- | --- | --- | --- | --- | --- | --- | --- | --- |
| dme03430 | Mismatch  repair | 17 | -0.739592638 | -2.194604841 | 0.000388802 | 0.013877119 | 0.011596789 | 2036 | tags=94%, list=17%, signal=78% | 34423/38492/40607/37290/40972/35364/32113/34842/34550/32763/35119/34892/36705/39746/35796/39654 |
| dme03440 | Homologous  recombination | 22 | -0.743128459 | -2.347771274 | 0.000426621 | 0.013877119 | 0.011596789 | 2500 | tags=91%, list=21%, signal=72% | 41839/44259/31044/41366/40972/35364/32113/41746/31565/43577/33507/34892/34565/39746/35236/48309/318579/37564/36136/35937 |
| dme03460 | Fanconi  anemia  pathway | 23 | -0.774869364 | -2.492096398 | 0.000429553 | 0.013877119 | 0.011596789 | 2441 | tags=96%, list=20%, signal=77% | 31044/35895/41366/40972/35364/36654/40965/32113/41231/38079/31565/43577/40438/31373/36705/35236/2768674/35796/318579/32608/36136/36199 |
| dme03030 | DNA  replication | 30 | -0.736465095 | -2.538225161 | 0.000494805 | 0.013877119 | 0.011596789 | 3041 | tags=93%, list=25%, signal=70% | 34423/37560/246459/38492/3772329/40607/37290/38942/31603/40972/35364/32113/34550/32763/36887/34892/35679/42553/39746/40973/41296/31449/39014/31934/44915/33213/43278/32323 |
| dme03420 | Nucleotide  excision repair | 33 | -0.619091504 | -2.171628131 | 0.000529661 | 0.013877119 | 0.011596789 | 3108 | tags=79%, list=26%, signal=59% | 35780/34423/37560/40429/36130/38492/39688/31441/3772329/31357/36598/40607/37290/40972/35364/36654/32113/41611/3772069/34550/32763/33294/34892/31373/39746/37414 |
| dme04624 | Toll and Imd  signaling  pathway | 70 | -0.613077608 | -2.519798733 | 0.000925069 | 0.020197348 | 0.016878459 | 994 | tags=29%, list=8%, signal=27% | 43283/39020/42253/41087/42791/35862/43256/43222/38419/2768679/36047/50126/32099/38858/43596/43598/39870/43599/39869/38408/37184 |
| dme03430 | Mismatch  repair | 17 | -0.739592638 | -2.194604841 | 0.000388802 | 0.013877119 | 0.011596789 | 2036 | tags=94%, list=17%, signal=78% | 34423/38492/40607/37290/40972/35364/32113/34842/34550/32763/35119/34892/36705/39746/35796/39654 |
| dme03440 | Homologous  recombination | 22 | -0.743128459 | -2.347771274 | 0.000426621 | 0.013877119 | 0.011596789 | 2500 | tags=91%, list=21%, signal=72% | 41839/44259/31044/41366/40972/35364/32113/41746/31565/43577/33507/34892/34565/39746/35236/48309/318579/37564/36136/35937 |
| dme03460 | Fanconi  anemia  pathway | 23 | -0.774869364 | -2.492096398 | 0.000429553 | 0.013877119 | 0.011596789 | 2441 | tags=96%, list=20%, signal=77% | 31044/35895/41366/40972/35364/36654/40965/32113/41231/38079/31565/43577/40438/31373/36705/35236/2768674/35796/318579/32608/36136/36199 |

**INT (compared with NINT)**

| **ID** | **Description** | **setSize** | **enrichmentScore** | **NES** | **pvalue** | **p.adjust** | **qvalues** | **rank** | **leading_edge** | **core_enrichment** |
| --- | --- | --- | --- | --- | --- | --- | --- | --- | --- | --- |
| dme03430 | Mismatch  repair | 17 | -0.739592638 | -2.194604841 | 0.000388802 | 0.013877119 | 0.011596789 | 2036 | tags=94%, list=17%, signal=78% | 34423/38492/40607/37290/40972/35364/32113/34842/34550/32763/35119/34892/36705/39746/35796/39654 |
| dme03440 | Homologous  recombination | 22 | -0.743128459 | -2.347771274 | 0.000426621 | 0.013877119 | 0.011596789 | 2500 | tags=91%, list=21%, signal=72% | 41839/44259/31044/41366/40972/35364/32113/41746/31565/43577/33507/34892/34565/39746/35236/48309/318579/37564/36136/35937 |
| dme03460 | Fanconi  anemia  pathway | 23 | -0.774869364 | -2.492096398 | 0.000429553 | 0.013877119 | 0.011596789 | 2441 | tags=96%, list=20%, signal=77% | 31044/35895/41366/40972/35364/36654/40965/32113/41231/38079/31565/43577/40438/31373/36705/35236/2768674/35796/318579/32608/36136/36199 |
| dme03030 | DNA  replication | 30 | -0.736465095 | -2.538225161 | 0.000494805 | 0.013877119 | 0.011596789 | 3041 | tags=93%, list=25%, signal=70% | 34423/37560/246459/38492/3772329/40607/37290/38942/31603/40972/35364/32113/34550/32763/36887/34892/35679/42553/39746/40973/41296/31449/39014/31934/44915/33213/43278/32323 |
| dme03420 | Nucleotide  excision repair | 33 | -0.619091504 | -2.171628131 | 0.000529661 | 0.013877119 | 0.011596789 | 3108 | tags=79%, list=26%, signal=59% | 35780/34423/37560/40429/36130/38492/39688/31441/3772329/31357/36598/40607/37290/40972/35364/36654/32113/41611/3772069/34550/32763/33294/34892/31373/39746/37414 |
| dme04624 | Toll and Imd  signaling  pathway | 70 | -0.613077608 | -2.519798733 | 0.000925069 | 0.020197348 | 0.016878459 | 994 | tags=29%, list=8%, signal=27% | 43283/39020/42253/41087/42791/35862/43256/43222/38419/2768679/36047/50126/32099/38858/43596/43598/39870/43599/39869/38408/37184 |
| dme03430 | Mismatch  repair | 17 | -0.739592638 | -2.194604841 | 0.000388802 | 0.013877119 | 0.011596789 | 2036 | tags=94%, list=17%, signal=78% | 34423/38492/40607/37290/40972/35364/32113/34842/34550/32763/35119/34892/36705/39746/35796/39654 |
| dme03440 | Homologous  recombination | 22 | -0.743128459 | -2.347771274 | 0.000426621 | 0.013877119 | 0.011596789 | 2500 | tags=91%, list=21%, signal=72% | 41839/44259/31044/41366/40972/35364/32113/41746/31565/43577/33507/34892/34565/39746/35236/48309/318579/37564/36136/35937 |
| dme03460 | Fanconi  anemia  pathway | 23 | -0.774869364 | -2.492096398 | 0.000429553 | 0.013877119 | 0.011596789 | 2441 | tags=96%, list=20%, signal=77% | 31044/35895/41366/40972/35364/36654/40965/32113/41231/38079/31565/43577/40438/31373/36705/35236/2768674/35796/318579/32608/36136/36199 |
